# Systematic Comparison of Retinal Organoid Differentiation from Human Pluripotent Stem Cells Reveals Stage Specific, Cell Line, and Methodological Differences

**DOI:** 10.1002/sctm.18-0267

**Published:** 2019-03-27

**Authors:** Carla B. Mellough, Joseph Collin, Rachel Queen, Gerrit Hilgen, Birthe Dorgau, Darin Zerti, Majed Felemban, Kathryn White, Evelyne Sernagor, Majlinda Lako

**Affiliations:** ^1^ Institute of Genetic Medicine Newcastle University Newcastle United Kingdom; ^2^ Centre for Ophthalmology and Visual Science Lions Eye Institute, University of Western Australia Perth Western Australia Australia; ^3^ Institute of Neuroscience Newcastle University Newcastle United Kingdom; ^4^ EM Research Services Newcastle University Newcastle United Kingdom

**Keywords:** Human embryonic stem cells, Induced pluripotent stem cells, Retina, Organoids, Tissue engineering

## Abstract

A major goal in the stem cell field is to generate tissues that can be utilized as a universal tool for in vitro models of development and disease, drug development, or as a resource for patients suffering from disease or injury. Great efforts are being made to differentiate human pluripotent stem cells in vitro toward retinal tissue, which is akin to native human retina in its cytoarchitecture and function, yet the numerous existing retinal induction protocols remain variable in their efficiency and do not routinely produce morphologically or functionally mature photoreceptors. Herein, we determine the impact that the method of embryoid body (EB) formation and maintenance as well as cell line background has on retinal organoid differentiation from human embryonic stem cells and human induced pluripotent stem cells. Our data indicate that cell line‐specific differences dominate the variables that underline the differentiation efficiency in the early stages of differentiation. In contrast, the EB generation method and maintenance conditions determine the later differentiation and maturation of retinal organoids. Of the latter, the mechanical method of EB generation under static conditions, accompanied by media supplementation with Y27632 for the first 48 hours of differentiation, results in the most consistent formation of laminated retinal neuroepithelium containing mature and electrophysiologically responsive photoreceptors. Collectively, our data provide substantive evidence for stage‐specific differences in the ability to give rise to laminated retinae, which is determined by cell line‐specific differences in the early stages of differentiation and EB generation/organoid maintenance methods at later stages.

**Significance Statement**

A greater understanding of the events controlling retinal organoid formation is essential in order to improve and standardize protocols for the production of physiologically relevant tissue across multiple cell lines. This study examines whether the methods used to generate embryoid bodies (EBs) from human pluripotent stem cells at the onset of differentiation, have any impact on the resulting retinal organoids. The data indicate that, during the early stages of differentiation, cell line‐specific differences dominate the variables that underline retinal organoid differentiation efficiency. In contrast, the EB generation method and maintenance conditions determine the later differentiation and maturation of retinal organoids.

## Introduction

An armory of protocols now exist that facilitate the generation of retinal tissue from human pluripotent stem cells. The production of photoreceptors from human stem cells was originally reported under two‐dimensional (2D) culture conditions which gave rise to photoreceptor precursor cells and some photoreceptors expressing mature photoreceptor markers 1, 2, 3, 4, 5. This was then achieved in three‐dimensional (3D) culture, which gave rise to developing photoreceptors within optic vesicle‐like structures that consisted of multiple retinal progeny 6 within distinct retinal laminae 7, 8, 9. This advance enabled the production of retinal tissue, which more closely mimicked the natural order of retinogenesis and microarchitecture of the native retina than 2D approaches had allowed. The late Yoshiki Sasai started a legacy with his pioneering work, which demonstrated that invagination of the optic vesicle and formation of the optic cup could be achieved from mouse and human embryonic stem cells (hESCs) in vitro, giving rise to the most complex retinal organoids achieved at the time 10, 11. Many reports which have followed these achievements are different permutations of these protocols 12, 13, 14, 15, 16, 17, 18, 19. In our previous study, we demonstrated that a 3D eye anlage could be generated in vitro, containing laminated retina alongside rudimentary lens and corneal tissue 8. This has also recently been reported by others using human induced pluripotent stem cells (hiPSCs) in a 2D culture system 20. Current retinal differentiation protocols can give rise to laminated tissue consisting of multiple phenotypes, but these are not fully morphologically or functionally mature. The confirmation of photoreceptor‐like electrophysiological properties including sensitivity of hESC/hiPSC‐derived photoreceptors to cGMP indicates that they contain some of the machinery necessary for phototransduction 8, 21, 22, 23, yet in only a couple of cases the infrequent observation of small immature developing outer segments has been observed, the most remarkable examples, with corresponding transmission electron microscopy (TEM) images of developing outer segment discs, by Zhong et al., Lowe et al., and Parfitt et al. 18, 24, 25. The ultimate goal, light sensitivity, has been demonstrated by our group and others 18, although comparable only to the earliest light responses recorded from the neonatal mouse retina, close to the period of eye opening 18, 26. Nonetheless, reports of the usefulness of this human tissue for disease modeling are already emerging 21, 22, 25, 27, 28, 29.

Heterogeneity within and between organoids is commonplace across protocols and intraline 26 and interlaboratory differences exist. Moreover, protocols that are successful in one hESC line may not work as efficiently in others, nor across multiple hiPSC lines 6, 26, and the epigenetic memory from the parental somatic cell may either help of hinder the differentiation of hiPSCs toward a particular lineage 12. There is a clear need for a greater understanding of the events controlling retinal organoid formation in order to improve upon and standardize protocols for the routine orchestration of useful retinal tissue across multiple cell lines.

We have previously reported protocols for the generation of photoreceptors from stem cells expanded on mouse embryonic fibroblasts (MEFs) and then differentiated under feeder‐free 2D or 3D culture conditions 3, 8. Following the adaptation of hESC and hiPSCs to feeder‐free and defined media conditions, we observed that the differentiation behavior of cells differed from our previous work. From our observations, we hypothesized that the initial phase of embryoid body (EB) formation was key to the overall success of the differentiation. This encouraged us to examine the retinal differentiation capacity of differentiating populations of hESC and hiPSCs, which had been generated using differing methods of EB formation.

Contraction of the actin‐myosin cytoskeleton is a critical effector of hESC death following cell dissociation, and the disruption of contraction by inhibition of Rho‐associated kinase (ROCK) or myosin light chain kinase can greatly increase cell viability 30, 31, 32. The application of ROCK inhibitor in differentiation methods adopting dissociation–reaggregation is thus commonplace 5, 11, 14, 33, 34. A neurogenic effect of ROCK inhibition (ROCK*i*) on stem cells has also recently been reported 35, 36, 37. Therefore, we incorporated ROCK*i* into our experiments to investigate any effects on differentiation outcome. Furthermore, to assess bioreactor‐like culture conditions, we included stationary versus shaking experimental groups into our investigation. Our data show cell line‐specific differences in the early differentiation of hESC and hiPSC to retinal organoids, which were accompanied by generation and maintenance method related differences during the later stages of differentiation.

## Materials and Methods

### hESC Culture and Differentiation

**Figure 1:**
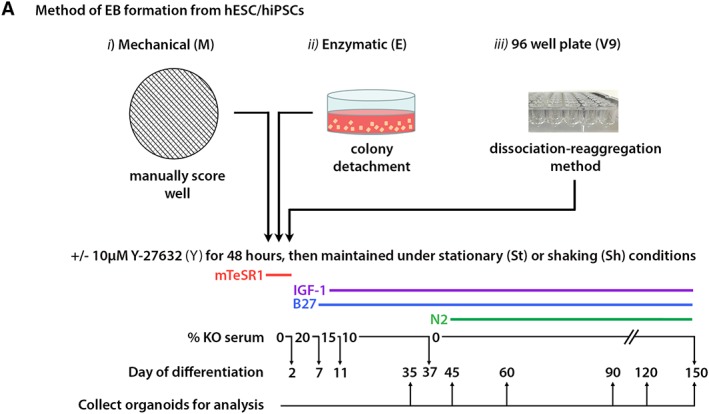
Schematic showing the various methods of embryoid body (EB) formation tested at the onset of differentiation. EB formation was initiated using either a mechanical (M), enzymatic (E), or dissociation–reaggregation approach in V‐shaped 96 well plates seeded with 9,000 (V9) cells per well plus or minus Y‐27632 (Y) in mTeSR1 medium for the first 48 hours, under either stationary (St) or shaking (Sh) conditions. Cells were then differentiated in the presence of IGF‐1 with B27 supplementation throughout and N2 supplementation from day 37. Serum supplementation was reduced to 0% by day 37. Organoids were collected at regular intervals for analysis.

One hESC line (H9, WiCell) one neonatal iPSC line (Neo1, male 38, 39) and one adult iPSC line (AD3, from a 37‐year‐old female 40) were adapted from MEF‐supported to feeder‐free culture conditions on 6 well plates coated with growth factor‐reduced Matrigel basement membrane matrix (GFRM; Corning, New York) in TeSR1 (Stem Cell Technologies, Vancouver, Canada) defined medium. Cells were fed daily and passaged every 5 days at a ratio of up to 1:10 using 0.02% versene EDTA (Lonza, Basel, Switzerland). Cells were then differentiated by initiating EB formation, followed by long‐term culture (up to 150 days). EBs were generated either by mechanical, enzymatic, or dissociation–reaggregation approaches (Fig. 1). Each method was tested in the presence or absence of ROCK inhibitor (Y‐27632, Chemdea, NJ) for the first 48 hours of differentiation, during the early phase of EB formation. All cultures were tested in parallel under either stationary (static, “St”) or shaking (“Sh”) conditions throughout differentiation. Shaking cultures were achieved by placing cells on an orbital shaker (30 rpm, Stuart) housed inside the incubator for the entire period of differentiation, or in the case of EBs generated via the dissociation–reaggregation method, from day 12 onward. Biological replicates were performed in triplicate (*n* ≥ 3) for all experimental conditions tested.

#### *Mechanical EB Formation (M)*

Cells were grown to 90% confluence on GFRM in TeSR1 media. For EB formation, cross hatches were made manually across the surface of the well using a sterile 1 ml pipette tip, cutting proliferative colonies into fragments approximately 1.4 mm^2^ (Fig. 1). Using a cell scraper, colony fragments were collected and transferred to 50‐ml tubes and allowed to settle with gravity. Two‐thirds of the supernatant was aspirated and the media refreshed with additional TeSR1 to the volume necessary for the number of petri dishes being seeded. The fragments collected from two 6‐well plates were transferred to one 90‐mm bacteriological petri dish (BD Biosciences, Berkshire, U.K.) in 14 ml TeSR1 media, either with or without ROCK*i* (10 μM Y‐27632). This resulted in the generation of floating colony fragments on day 0 of differentiation (Fig. 1). TeSR1 media was changed after 48 hours to retinal differentiation media (10 ml per petri dish).

#### *Enzymatic EB Formation (E)*

Cells were grown to 90% confluence. TeSR1 media was removed and the cells washed with phosphate‐buffered saline. Colony detachment solution (1 mg/ml collagenase with 0 .5mg/ml dispase in Dulbecco's modified Eagle's medium [DMEM]:F12) was then added to each well (1 ml per well) and the cells incubated at 37°C for 30–45 minutes. Detached colonies from each plate were transferred to a 50 ml Falcon tube and the detachment solution was diluted by adding a 1:5 mix of DMEM:F12 and TeSR1, bringing the volume from each plate to 30 ml. The colonies were allowed to settle into a loose pellet with gravity, the supernatant removed, then the pellet washed with 30 ml of 1:5 DMEM:F12/TeSR1. Following the settlement of colonies with gravity once more, colonies were resuspended in 12 ml TeSR1 media and transferred to a 90 mm petri dish, either with or without ROCK*i* (10 μM Y‐27632). Media was changed after 48 hours to 10 ml retinal differentiation media.

#### *Dissociation–Reaggregation EB Formation (V9)*

This approach is a modified simplified version of the protocols published by Eiraku et al. and Nakano et al. 11, 41. Cells were harvested at 90% confluence. hESCs/hiPSCs were dissociated to a single cell suspension by treatment with Accumax (1 ml per well) for up to 3 minutes. TeSR1 was added to each well and dissociated cells were collected and spun at 1,000 rpm for 5 minutes. Cell pellets were resuspended in TeSR1 medium containing 10 μM Y‐27632, counted in a hemocytometer, and reaggregated by seeding 9,000 cells per well in 100 μl into low adhesion Sumilon PrimeSurface 96 well plates with V‐bottomed conical wells (Sumitomo Bakelite, Osaka, Japan) or V‐bottomed 96 well plates coated with 0.5 wt% Lipidure‐CM5206 (amsbio). After 48 hours, the media was changed to retinal differentiation media. Media was changed every 3 days. On day 12, the resulting EBs were collected using a sterile disposable plastic Pasteur pipette and transferred into a low attachment 6 well plate (Corning) or 9 cm petri dish.

Cultures were differentiated in retinal differentiation medium comprising of ventral neural induction media supplemented with recombinant human IGF‐1 (Sigma, Hartfordshire, U.K.) as previously described 3, 8.

### Immunocytochemistry and TEM

Organoids were fixed over days 35–150 (5–21 weeks) of differentiation and immunocytochemistry performed on cryostat sections as previously described 3, 8. Sections were reacted against a panel of retinal, lens, and corneal‐specific antibodies, listed in Supporting Information Table S1. At least five structures from each time point across each experimental group were immunostained. Images were obtained using a Zeiss Axio Imager.Z1 microscope with ApoTome.2 accessory equipment and AxioVision or Zen software. For TEM tissues were fixed in glutaraldehyde and processed by the Electron Microscopy Research Service at Newcastle University. Human embryonic and fetal samples were provided by the Human Developmental Biology Resource (http://hdbr.org) under ethics permission (09/H0906/21).

### Array‐Based Gene Expression Analysis

RNA was extracted from retinal organoids (RNeasy Plus, QIAGEN, Manchester, U.K.), cDNA synthesized (Reverse Transcription Master Mix, Fluidigm, San Francisco, CA) and preamplified (PreAmp Master Mix, Fluidigm). Preamplified cDNA and TaqMan Assays (Thermo Fisher Scientific, MA) were loaded onto a primed 96.96 integrated fluidic circuit (Fluidigm) using a HX controller (Fluidigm). qPCR and data collection was then performed using a Biomark HD (Fluidigm). For TaqMan assays used, see Supporting Information Table S2. CT values were calculated using the Fludigm Real‐time Analysis software and the expression values extracted using HTQPCR bioconductor package version 1.30. To assess the success of the experiment, the number of genes detected per cell along with the total expression levels was analyzed. Samples were filtered if the total raw expression value was less than 500, or fewer than 60 genes were, or no housekeeping genes were expressed. The filtered data was normalized with the normalizeCtData() function in the HTQPCR package using the housekeeping genes. Technical replicates were condensed by taking the mean expression for each gene. Principal component analysis (PCA) of all samples was used to visualize the distance between all samples. The data was subset to look specifically at samples at week 5 and from day 120 and above. PCA was performed to visualize the samples at both of these time points.

The Wilcoxon test was used to look for significant differences between embryonic/fetal retinal and retinal organoids generated from hESCs and hiPSCs with the different methods for samples after 5 weeks of differentiation. *p* values were adjusted using Bonferroni correction and an adjusted *p* value threshold of <.05 was taken as significant. The total numbers of genes that were significantly different from the reference samples, and the numbers of 32 marker genes known to be expressed in rod, cone, and photoreceptors were calculated and plotted. The similarity in expression between human embryonic/fetal retina and retinal organoids generated from hESCs and hiPSCs differentiated in either static or shaking conditions at day 120 and above was compared. The mean expression for each of the 32 marker genes human embryonic/fetal samples was calculated and compared with individual samples by computing the Pearson correlation coefficient. These values were then plotted as a boxplot to visualize the similarities between each set of samples and the human embryonic/fetal samples. The Wilcoxon test with Bonferroni multiple test correction was applied to the data compare expression of samples in either static or shaking conditions and human embryonic/fetal retina samples. Known marker genes with a significant adjusted *p*‐value of below .05 were plotted as a heatmap.

### Electrophysiology

Twenty‐four hours prior to electrophysiological recordings, 9‐cis‐retinal (10 nM; Sigma–Aldrich, U.K.) was added to the incubation medium. Organoids were transferred to 34°C artificial cerebrospinal fluid (aCSF) containing the following (in mM): 118 NaCl, 25 NaHCO_3_, 1 NaH_2_ PO_4_, 3 KCl, 1 MgCl_2_, 2 CaCl_2_, 10 glucose, 0.5 l‐glutamine, and 0.01 9‐cis‐retinal. Organoids were opened longitudinally and placed, with the presumed RGC layer facing down on the electrodes, onto the 4,096 channel multielectrode array (MEA), flattened with a translucent polyester membrane filter (Sterlitech Corp., Kent, WA). The organoids were allowed to settle for at least 2 hours. Recordings were performed on the BioCam4096 MEA platform with BioChips 4096S+ (3Brain GmbH, Lanquart, Switzerland), integrating 4,096 square microelectrodes in a 64 × 64 array configuration.

Light stimuli were projected as described previously 42. Broad white (high photopic) light pulses (WLP, 200 ms, 217 μW/cm^2^ irradiance, 1 Hz) were flashed for 5 minutes onto the organoids following recording spontaneous activity in the dark for 5 minutes. We also used sustained broad blue light stimulation (SBL, 2 minutes darkness, 2 minutes SBL, 2 minutes darkness), same irradiance as WLP), to evoke responses from intrinsically photosensitive RGCs (ipRGCs). The drugs cGMP (8‐bromoguanosine 3′,5′‐cyclic monophosphate, Sigma–Aldrich, MO) and GABA (Tocris, U.K.) were puffed in the recording chamber (final concentrations: 100 μM and 125 μM, respectively) and activity was recorded continuously for 4 minutes, starting at 2 minutes before the puff.

To reliably extract spikes from the raw traces we used a quantile‐based event detection 43. Single‐unit spikes were sorted using an automated spike sorting method for dense, large‐scale recordings 44. Statistical significance (unpaired *t* test) and firing rate analyses were evaluated by using MATLAB (Mathworks, MA) and Prism (GraphPad, CA). RGCs were considered responsive if they show at least 25% increase or decrease in spiking activity during 30 seconds after WLP onset compared with a similar time window before the light was turned on. For each cell, all spikes occurring during these two time windows were counted and the mean % change (±SEM) in activity between windows was calculated. All RGCs respond to WLP. To single out ipRGCs, we performed additional analysis of their responses to SBL, taking into account that photoreceptor‐driven responses are relatively transient, and therefore stop after a while, whereas intrinsic light responses in ipRGCs are sustained (and generally have a slower onset). Hence, we classified RGCs as ipRGCs if they still exhibited significantly higher firing rate 30–60 seconds after the onset of the SBL stimulus. All other RGCs were analyzed for the WLP protocol (photoreceptor‐driven responses).

## Results

### Early Stages of Embryoid Body Formation from hESCs and hiPSCs Using Differing Protocols

**Figure 2:**
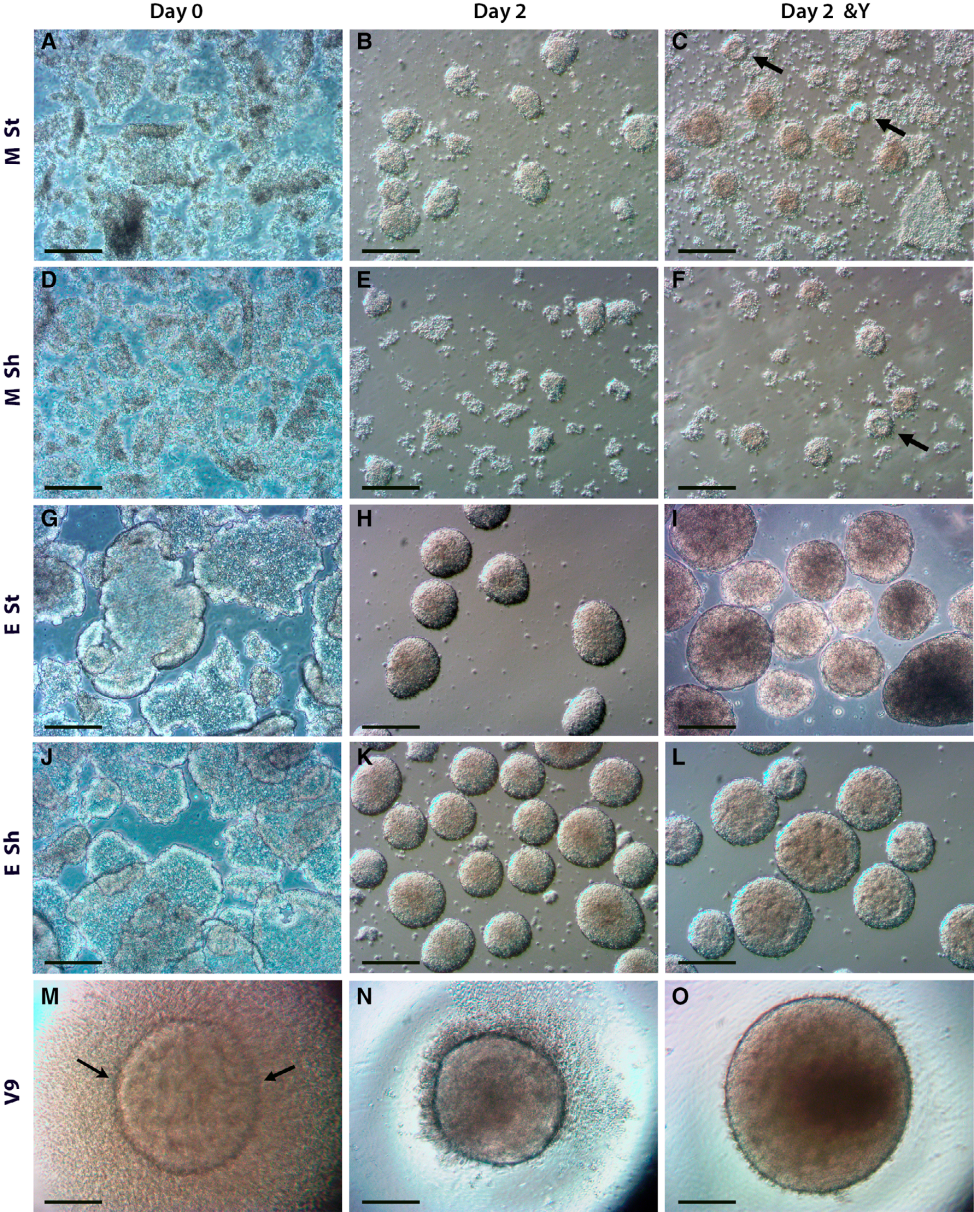
Phase micrographs showing the morphology of cultures generated by different methods during the first 48 hours of differentiation. Proliferative cultures on day 0 collected by mechanical means **(A, D)** were observed as square‐shaped floating fragments of colonies which, by day 2 **(B, C, E, F)**, gave rise to small EBs in cultures that still contained cell debris. (D–F): Fewer embryoid bodies (EBs) were formed under shaking conditions following mechanical EB formation. **(G, J):** Cultures collected by enzymatic means on day 0 consisted of larger fragments in comparison to mechanical cultures, and were more phase‐bright with softer edges. **(H, I, K, L):** By day 2, similarly sized enzymatically derived EBs were generated under both stationary (St) and shaking (Sh) conditions with the addition of Y‐27632 (Y) resulting in EBs of increased size. Less cellular debris was observed in enzymatically generated cultures during the first 48 hours. **(M–O):** Micrographs showing V9 EBs formed by dissociation–reaggregation on (M) day 0 and (N, O) after 48 hours in the absence (N) or presence (O) of Y‐27632. (O): V9&Y EBs were notably larger. All scale bars = 200 μm.

Proliferative cultures were processed to initiate EB formation using either a mechanical (M), enzymatic (E), or dissociation–reaggregation approach (V9; Fig. 1). Cultures collected by mechanical means appeared as square‐shaped colony fragments at the onset of differentiation (Fig. 2A, 2D), whereas enzymatically collected fragments had softer, more phase‐bright edges (Fig. 2G, 2J). After 48 hours, small (<150 μm in diameter) EBs had formed within all mechanical cultures (Fig. 2B, 2C, 2E, 2F), with residual cell debris observed in the media under these conditions. ROCK inhibition of mechanical cultures by supplementation with Y‐27632 (M&Y, both St and Sh) gave rise to EBs which appeared as spherical formations of neuroepithelium (Fig. 2C, 2F, arrows). Fewer EBs were observed in shaking cultures at this time point (Fig. 2E, 2F). In comparison to mechanical cultures, EBs formed via the enzymatic method measured <200 μm in diameter after 48 hours (Fig. 2H, 2I, 2K, 2L), and very little cell debris was observed. The addition of Y‐27632 to enzymatic cultures resulted in larger (up to 350 μm), less phase‐bright EBs, which appeared to have small rosette formations in the interior (Fig. 2I, 2L). In general, EBs cultured in the absence of Y‐27632 appeared homogenous and phase‐bright during the first 48 hours, whereas ROCK*i* resulted in early organization of EBs into neuroepithelial‐like formations (either in vesicular or rosette formations). Dissociation–reaggregation of cells into 96 well plates (“V9,” 9,000 cells per well) allowed almost immediate aggregation of cells at the bottom of the well, surrounded by some cellular debris, forming EBs of 400 μm diameter (Fig. 2M). V9 EBs were consistently larger (diameter increased to >600 μm) if generated in the presence of Y‐27632 (Fig. 2N, 2O). The effect of ROCK*i* was additive; EBs increased in size further if the concentration of Y‐27632 applied to cultures during the first 48 hours was doubled (Supporting Information Fig. S1). The diameter of EBs generated enzymatically without ROCK inhibition was <200 μm, whereas the addition of 10 μM Y‐27632 increased EB diameter to 200–350 μm, and treatment with 20 μM Y‐27632 increases EB diameter to >400 μm.

### Neuroepithelial Formation Under Differing Methods

Retinal organoids exhibit a typical morphology that is clearly identifiable during in vitro culture, namely, phase‐bright spherical EBs with layers of thick peripheral neuroepithelium (Supporting Information Fig. S2). This is better exemplified following sectioning on week 5, where these layers can be clearly identified as inner and outer neuroblastic layers (Supporting Information Fig. S3A, S3B). The overall morphology of organoids within cultures after week 5 varied greatly depending on the method of EB formation used at the onset of differentiation (Supporting Information Fig. S2A–S2I). Of note, shaking mechanical cultures gave rise to organoids with significantly flattened edges (Supporting Information Fig. S2A) consisting of one dense peripheral layer (Supporting Information Fig. S3C, S3D), whereas 96‐well plate‐generated cultures gave rise to organoids which were folded and could become highly convoluted (Supporting Information Fig. S2G, S2H) with a main body containing neural rosettes (Supporting Information Fig. S3E–S3G, asterisks). The generation of organoids was particularly difficult under enzymatic shaking culture conditions with ROCK*i* (E&Y, Sh); all cell lines failed to generate retinal organoids using this approach. Furthermore, the hESC line did not respond well to retinal differentiation under any dissociation–reaggregation conditions tested, forming instead EBs which became folded and developed fine extensions growing from the main body, an effect which was more pronounced under shaking conditions (Supporting Information Figs. S2G, S3E–S3G, arrows). The Neo1 hiPSC line was unable to form retinal organoids in V9 cultures, whereas increasing the initial seeding density to 12,000 cells per well (V12) permitted retinal organoid formation under stationary culture conditions only (Supporting Information Figs. S2H, S3G). The AD3 hiPSC line in general showed a greater ability to self‐organize into retinal neuroepithelium under all conditions tested, with the exception of the E&Y, Sh approach. Furthermore, the overall morphology of organoids in a culture could be altered significantly by whether these were shaken or not, as in the case of H9's generated in 96 well plates, or whether cells were reaggregated in a U‐shaped or V‐shaped well, as in the case of AD3s (Supporting Information Fig. S4). Neuroepithelium was observed in organoids generated from hESCs using both U‐shaped and V‐shaped 96 well plates if kept under stationary conditions (Supporting Information Fig. S4A, S4B), but not under shaking conditions (Supporting Information Fig. S4E, S4F). Using the AD3 hiPSC line, U‐shaped wells gave rise to organoids with clearly identifiable retinal neuroepithelium under both stationary and shaking conditions (Supporting Information Fig. S4C, S4G), whereas those generated using V‐shaped wells displayed thinner, more convoluted neuroepithelium (Supporting Information Fig. S4D, S4H). Although retinal organoids were able to form at different frequencies under almost all conditions tested within 5 weeks, these were most consistently achieved using a mechanical stationary approach (Supporting Information Fig. S2A–S2C, top row and Supporting Information Fig. S3A, S3B). Together, these observations show cell line‐specific and method‐associated differences in the ability of hESC and hiPSC to form phase‐bright spherical organoids with layers of thick peripheral neuroepithelium.

### Gene Expression Analysis Reveals Cell Line‐Specific Differences to Generate Retinal Organoids at Week 5 of Differentiation

To investigate potential differences between cell lines and methods used, a Taqman qRT‐PCR based array was compiled (Supporting Information Table S2) including key marker genes characterizing various retinal cell types. Retinal organoids generated from the pluripotent stem cells lines including hESC‐H9 (annotated as H), adult fibroblast derived hiPSC‐AD3 (annotated as A), and neonatal fibroblasts derived hiPSC‐Neo1 (annotated as N) were compared with eight samples of embryonic eyes obtained from 4.6 to 8 postconception week (PCW; annotated as hEye), 21 samples of fetal neural retina encompassing 8–18 PCW (annotated as hRet), and undifferentiated hESCs (annotated as hESC). Different methods of retinal organoid formation were compared under static (St) and shaking (Sh) conditions.

**Figure 3:**
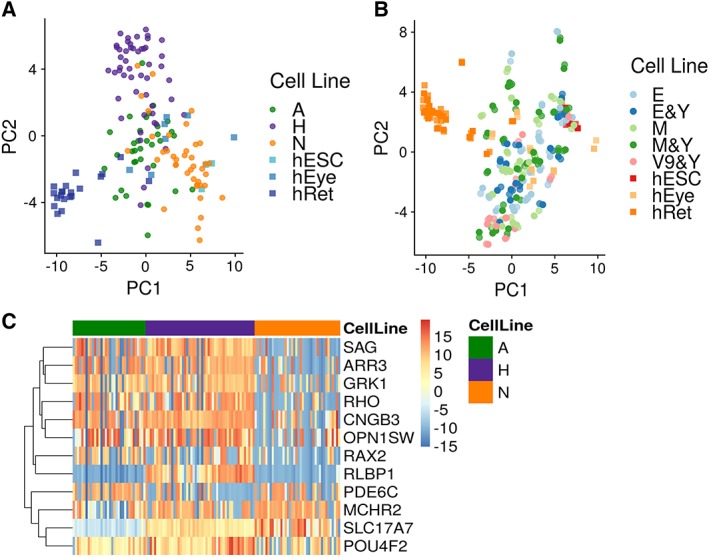
Gene expression analysis reveals that retinal organoids cluster by cell type and not by method at week 5 of differentiation. **(A):** Principal component analysis (PCA) analysis showing clustering by cell type. All three pluripotent cell lines, undifferentiated human embryonic stem cell and human embryonic eye, and fetal retinal samples are indicated by a different color. **(B):** PCA analysis showing no clustering by method of retinal organoid formation; all methods are indicated by different colors. **(C):** Heatmap showing differentially expressed genes between retinal organoids generated from all three pluripotent stem cell lines at week 5 of differentiation, *p* < .05.

PCA analysis of gene expression data indicated that at week 5, the retinal organoid clustered more strongly by cell type (Fig. 3A) than by method (Fig. 3B). The Wilcoxon test was carried out to identify genes that were differentially expressed between week 5 retinal organoids from all three pluripotent stem cell lines (Supporting Information Table S3 and Fig. 3C). This analysis indicated line specific differences; however, no significant differences in gene expression were observed between different methods used for retinal organoid formation at this time point (Fig. 3B). Addition of ROCK*i* or static versus shaking conditions had no impact on gene expression at this stage (Fig. 3B).

**Figure 4:**
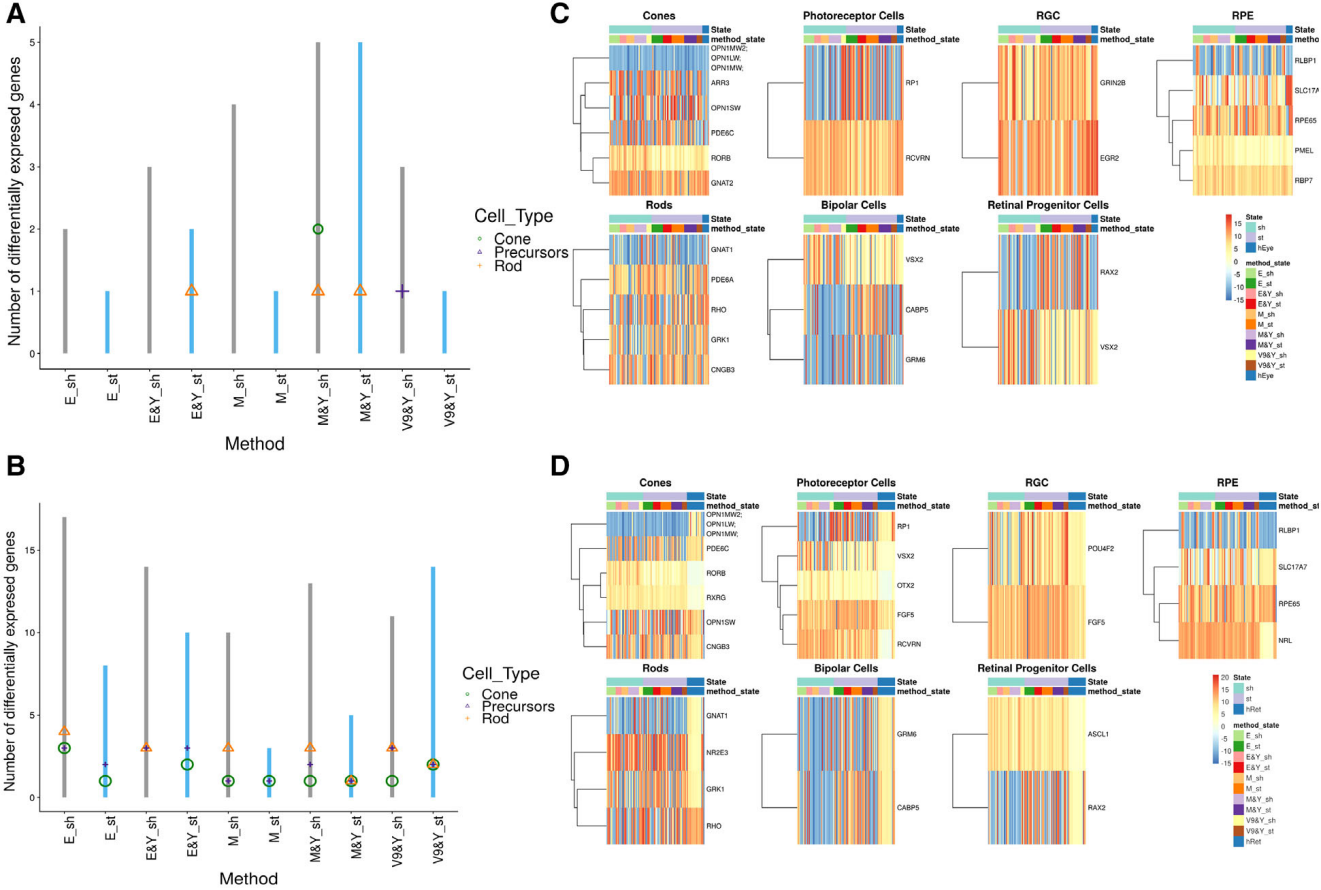
Retinal organoids are more similar to human embryonic eye samples than fetal/adult neural retina at week 5 of differentiation. **(A):** Schematic chart showing the number of differentially expressed genes between retinal organoids generated with different methods and human embryonic eye samples (hEye). **(B):** Schematic chart showing the number of differentially expressed genes between retinal organoids generated with different methods and human fetal retina samples (hRet). The photoreceptor, cone, and rod markers are shown by different shape and color (A, B). **(C):** Heatmaps based on major retinal cell types showing differentially expressed genes between retinal organoids generated with various methods and hEye samples, *p* < .05. **(D):** Heatmaps based on major retinal cell types showing differentially expressed genes between retinal organoids generated with various methods and hRet samples, *p* < .05.

To investigate whether the expression of key retinal markers at week 5 retinal organoids was closer to embryonic or fetal retina, differentially expressed genes between each EB generation method (all cell lines pooled together) and hEye and hRet samples were identified (Fig. 4A, 4B and Supporting Information Table S4). This analysis indicated that across the methods, there were less differentially expressed genes when week 5 retinal organoids samples when compared with hEye (Fig. 4A) than to hRet samples (Fig. 4B), suggesting that at this stage week 5 organoids are closer in expression profile to the embryonic eye. One of the methods, namely mechanical under static conditions (M_St) showed the lowest number of differentially expressed genes when compared with both hEye and hRet (fetal eye) samples (Supporting Information Table S4 and Fig. 4C). Overall, the retinal organoids made with enzymatic, mechanical or 96v shaped well plates with Y26732 inhibitor under static conditions, showed the least significant changes when compared with hEye samples (Fig. 4A), suggesting that for week 5 of differentiation these could be the best methods for acquiring a gene expression pattern that mimics eye development between 4.6 and 8 PCW.

Comparison of retinal organoids samples at week 5 of differentiation to hRet samples showed a larger number of genes to be differentially expressed, as expected, due to differences in developmental times. Nonetheless, the genes that were downregulated between week 5 retinal organoids and fetal retina samples belonged to mature cone and rod markers (*OPN1MW, OPN1LW, GNAT1*), bipolar cells (*GRM6, CABP5*), cilia marker (*RP1*) and melanopsin‐photosensitive ganglion cells (*OPN4*), which develop later during retinogenesis 45 and suggest that longer differentiation is needed to achieve appropriate expression of mature retinal cell markers (Supporting Information Table S4 and Fig. 4D). Notwithstanding, retinal organoids generated via mechanical means with and without Y26732 under static conditions showed the least differences in gene expression patterns when compared with hRet samples, again indicating they may be the most appropriate for achieving a transcriptional profile akin to neural retina.

**Figure 5:**
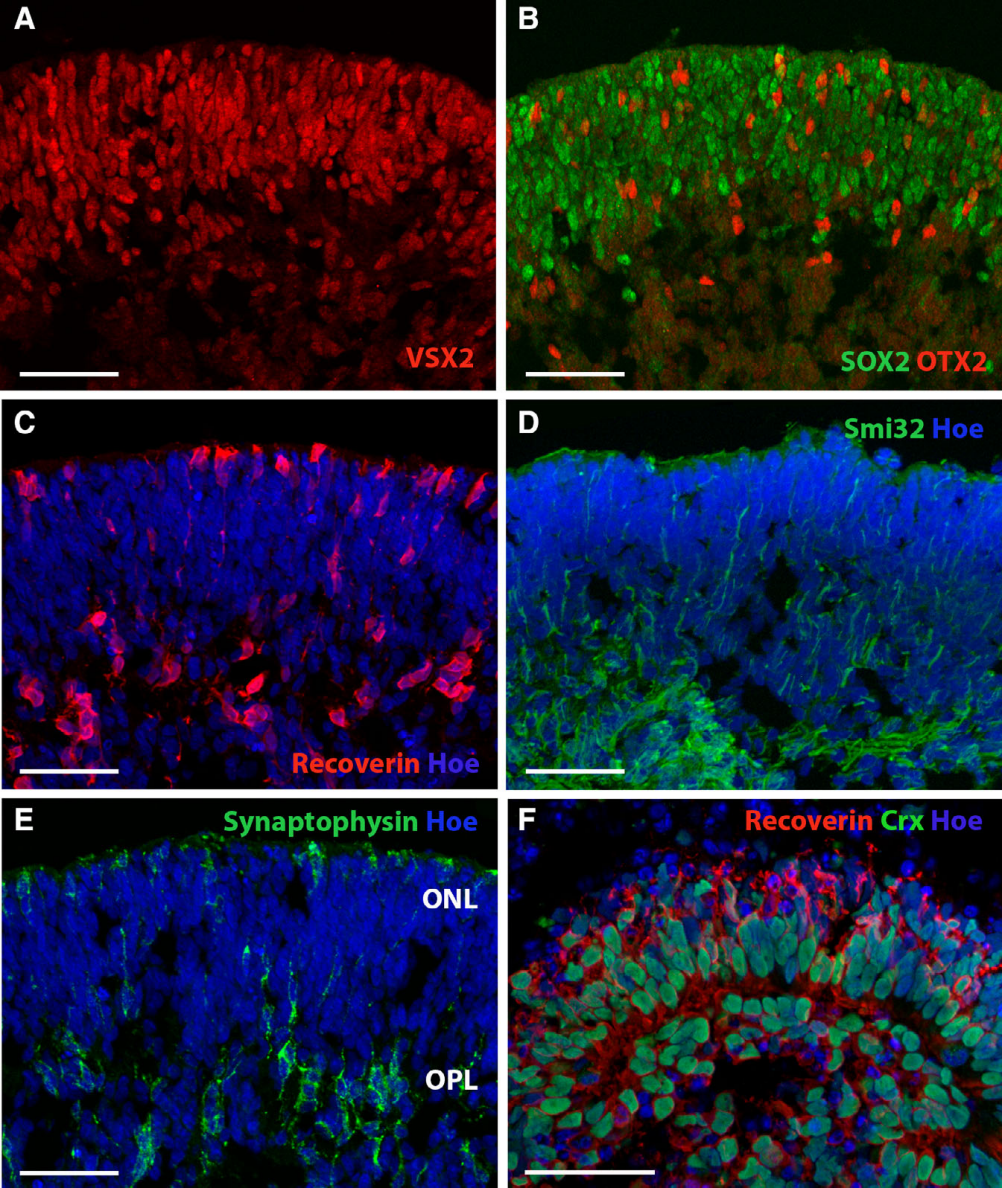
Immunohistochemistry on human embryonic stem cell‐derived organoids revealed the highest frequency of developing retinal tissue (78.5% of embryoid bodies) in cultures derived using the “M&Y_St” method on week 5 of differentiation. **(A–E):** Immunohistochemical screening of retinal organoids on week 5 of differentiation revealed developing neuroepithelial tissue containing (A) retinal progenitor cells stained with VSX2 and (B) Sox2, as well as photoreceptors stained with (B) OTX2 and (C) recoverin at the apical aspect, (D) retinal ganglion cells stained with Smi32 at the basal aspect, and (E) evidence of synaptogenesis in the developing outer nuclear layer (ONL) and outer plexiform layer as indicated by synaptophysin immunoreactivity. Neural retinal formation was observed at the highest frequency in cultures generated using a “M&Y_St” method and retinal tissue continued to develop over time in culture. **(F):** Retinal photoreceptors in the ONL of organoids attain morphological features of maturing cells, here shown at week 21 of differentiation, stained with Crx and Recoverin. Scale bars = 50 μm.

**Figure 6:**
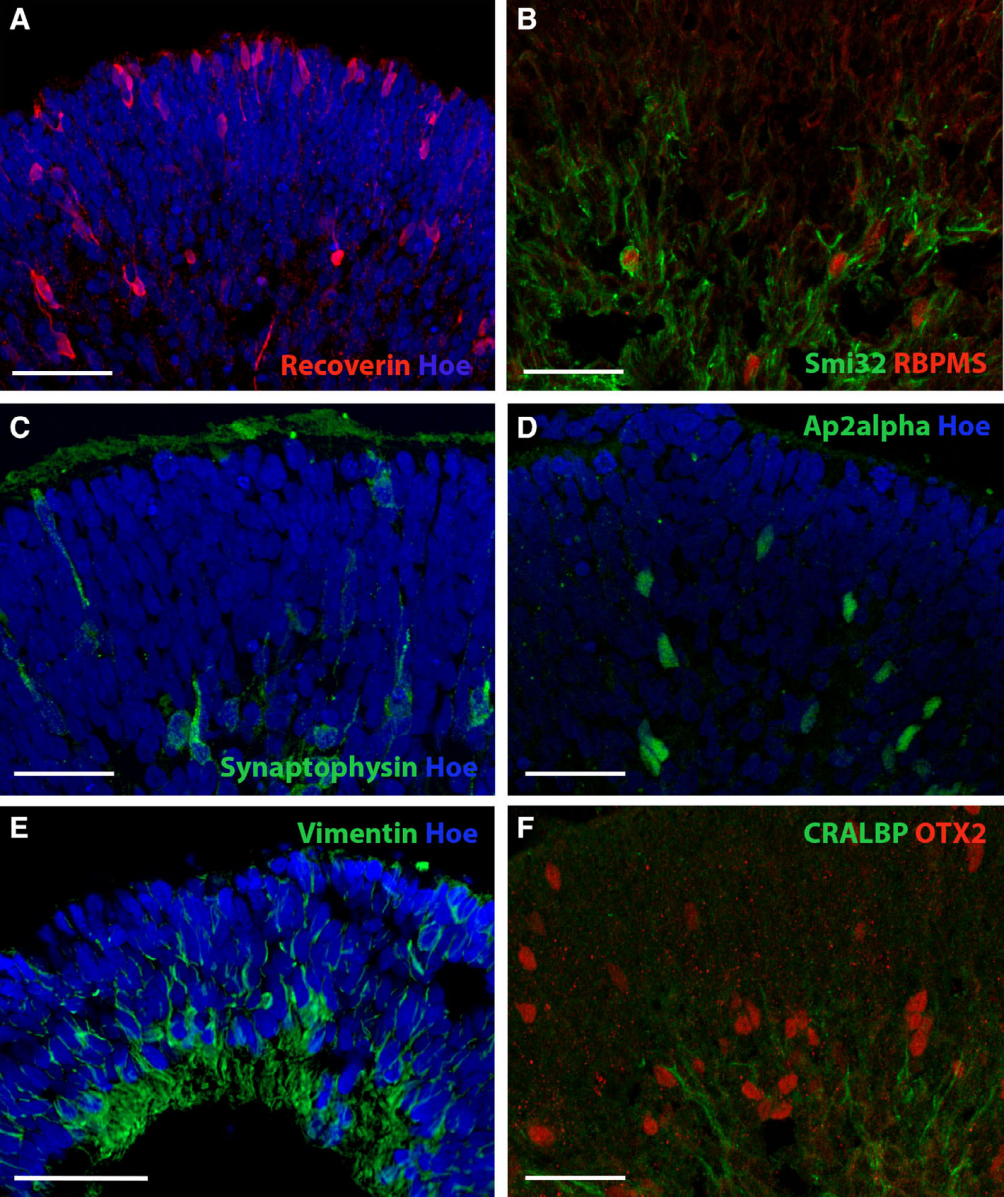
Characterization of human induced pluripotent stem cell‐derived organoids revealed a high frequency of developing retinal tissue in cultures derived using the “M&Y_St” method on week 5 of differentiation. Developing retinal neuroepithelium in **(A, B)** 12.5% of Neo1‐derived and **(C–F)** 94.4% of AD3‐derived organoids, demonstrating the presence of (A, F) photoreceptors (Recoverin, OTX2), (B) retinal ganglion cells (Smi32, RBPMS), (C) synaptic connections, (D) interneurons (Ap2α), and (E, F) Müller glial cells (Vimentin, CRALBP). Scale bars = 50 μm.

Following this transcriptional analysis, we performed immunohistochemistry (IHC) with retinal cell markers (Figs. 5, 6) which showed that at this stage, the highest frequency of developing laminated retinal tissue across all cell lines tested was observed in cultures derived using the “M&Y_St” method (78.5% of EBs in hESCs, 94.4% of EBs in AD3, and 12.5% of Neo1 EBs; Figs. 5A–5E, 6C–6F). Within these organoids, retinal progenitor cells immunopositive for VSX2 and SOX2, as well as photoreceptors stained with OTX2 and Recoverin were observed at the apical aspect of developing neuroepithelial tissue (Figs. 5A–5C, 6A, 6F). Developing retinal ganglion cells with Smi32‐immunopositive processes 46, 47 (Figs. 5D, 6B) and RBPMS‐immunopositive nuclei 48 (Fig. 6B) were located at the basal aspect. Horizontal cells detected by cellular immunoreactivity for Ap2α were also present (Fig. 6D). Furthermore, there was evidence of synaptogenesis in the developing outer nuclear layer (ONL) and outer plexiform layer as indicated by synaptophysin immunoreactivity (Figs. 5E, 6C) and beginning of Müller glia formation as shown by vimentin and CRALBP (RLBP1) staining throughout the retinal organoids (Fig. 6E, 6F). Mechanical stationary cultures initiated in the absence of Y‐27632 were also able to develop into retinal organoids with laminated retina containing Recoverin‐positive photoreceptors, however, these cultures gave a lower overall yield (in the best case, 73.3% of EBs sampled at week 5 in AD3‐hiPSC‐derived cultures).

In summary, our transcriptional and IHC analysis suggests the mechanical method of EB formation supplemented with Y27632 for the first 48 hours of differentiation and accompanied by stationary organoid maintenance conditions (M&Y_St) generate the best retinal organoids, which contain all key retinal cell types with gene expression that is closer to developing fetal retina.

### Mechanical EB Generation and Supplementation of Culture Media with Y27632 Under Static Conditions Generates Retinal Organoids Containing Electrophysiologically Responsive Photoreceptors

**Figure 7:**
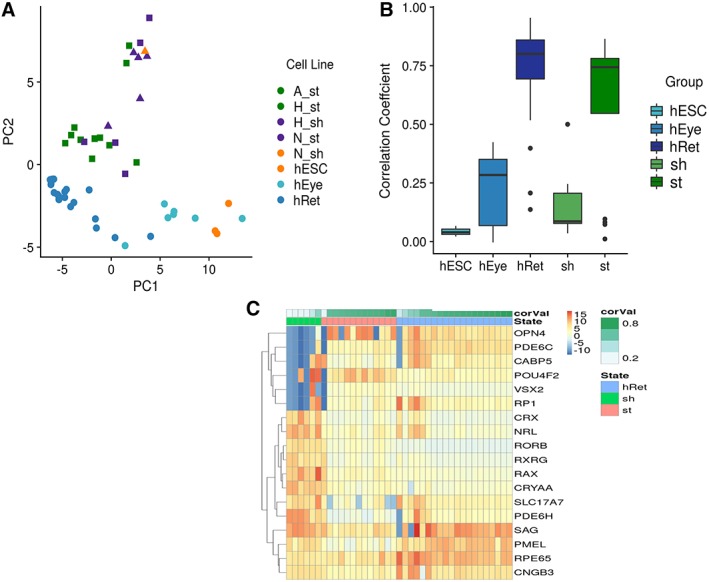
Retinal organoids generated under static conditions are more similar to fetal retina samples at later stages of differentiation (19–21 week of differentiation). **(A):** Principal component analysis showing clustering by condition, shaking, and static conditions are shown with different shapes. **(B):** Pearson correlation between retinal organoids and human embryonic eyes and fetal retinal (hRet) samples. The range of values within groups is shown in the boxplots. **(C):** Heatmap showing differentially expressed genes between retinal organoids generated under static and shaking conditions and hRet samples, *p* < .05.

The stability of retinal organoids was monitored by bright phase and fluorescence microscopy until the 19th–21st week of differentiation. Retinal organoids generated with the mechanical and 96 V‐shaped plates from all three cell lines survived past the 19th week of differentiation under both static and shaking conditions. Retinal organoids made from both hiPSC lines under the enzymatic conditions with the shaking or static methods degraded before the 19th week; however, organoids made with this method from hESC under static conditions were able to develop past this time point and were included in the qRT‐PCR array analysis for this later differentiation time point (Supporting Information Table S2). PCA analysis of gene expression data indicated that retinal organoids collected during the 19th–21st week of differentiation clustered more strongly by the condition (static or shaking) than by cell line (Fig. 7A). To understand whether organoids generated with the static or shaking conditions were more similar to embryonic eye (hEye) or human fetal retinal samples (hRet), Pearson correlation between the organoid samples, hEye, hESC samples, and hRet was calculated based on the mean expression of all 96 marker genes included in the qRT‐PCR array. This analysis (Fig. 7B) shows that retinal organoids generated under static conditions are much more similar to hRet samples than those generated under shaking conditions, indicating an advanced stage of maturation. This was further corroborated by comparison of gene expression profiles between retinal organoids generated under static and shaking conditions and hRet samples, which showed a higher expression of photoreceptor precursor markers (for example *CRX, NRL*, Supporting Information Table S5 and Fig. 7C) in retinal organoids generated under shaking when compared with those from stationary. Collectively, these data indicate that shaking conditions are more likely to enhance the generation of photoreceptor precursors; however, the overall expression profile of retinal organoids cultured under static conditions is closer to fetal/adult neural retina samples, suggesting that the latter represents a useful method for generating retinal organoid samples that mimic more closely the developing human retina.

These findings were corroborated by the IHC analysis, which showed that static cultures (generated via mechanical and 96 well plate methods) across all cell lines contained impressive areas of laminated retina with aligned Recoverin‐immunopositive photoreceptors and some RPE by week 12 of differentiation. In contrast, shaking cultures by week 13 consistently displayed large disorganized patches of nonaligned immature photoreceptors with apically positioned Smi32 processes, however, the overall internal architecture was disorganized. By week 17, large clusters of photoreceptors were observed in the statically maintained cultures, some of which formed rosettes, whereas others were located at the apical aspect of laminated retinal neuroepithelium neighboring basally positioned retinal ganglion cells (data not shown). The most advanced retinal neuroepithelium with photoreceptors developing with opposing polarity to retinal ganglion cells, was observed in cultures generated using the M&Y_St method. Photoreceptors in the ONL of hESC‐derived and hiPSC‐derived retinal organoids derived using the M&Y_St approach developed mature morphological features and were immunoreactive for mature markers (Supporting Information Fig. S5). In addition to being immunopositive for the pan‐photoreceptor marker Recoverin (Supporting Information Fig. S5A, S5D), by week 21 of differentiation photoreceptors were also immunopositive for cone‐specific Opsin Blue, Bassoon (Supporting Information Fig. S5B), and rod‐specific Gαt1 (Supporting Information Fig. S5C). Transmission electron microscopy of “M&Y_St” retinal organoids on week 24 of differentiation revealed radially aligned photoreceptors displaying mitochondrial‐rich inner segments (Supporting Information Fig. S5E–S5H). A connecting cilium extended from each inner segment, and this was connected to rudimentary outer segments (Supporting Information Fig. S5E–S5I). Observation of developing outer segments at high magnification revealed the appearance of membrane discs which were stacked on top of each other (Supporting Information Fig. S5J, S5L), with some outer segments appearing disorganized (Supporting Information Fig. S5I, S5K). Depending on orientation, the edge of developing membrane discs was observed to be capped by circular membrane structures (most clearly visible in Supporting Information Fig. S5I, S5J).

To investigate the function of laminated retinal epithelium generated under static conditions, in vitro extracellular large‐scale high‐density multielectrode recordings (BioCam, 3brain AG, Switzerland) of retinal ganglion cells (RGCs) were carried out, as described in our previous study 26, on samples generated from “M&Y_St” conditions. Many presumed RGCs at week 21 of differentiation exhibited at least 25% increase (37/164 RGCs) or decrease (40/164 RGCs) in spiking activity when exposed to a pulsed high intensity stimulus (WLP, Supporting Information Fig. S6A, S6B). These response types could come from premature ON and OFF RGCs. To ensure that the responses being observed were indeed photoreceptor driven, we used sustained blue light (SBL) to separate intrinsically photosensitive RGCs (ipRGCs) from photoreceptor‐driven RGCs and found many activated ipRGCs (48/164 RGCs) within organoids (Supporting Information Fig. S6C). Puffing 8‐br‐cGMP, a membrane permeable analog of cGMP, in the recording chamber triggers Na + influx and depolarises photoreceptors, thus mimicking the dark current. RGCs responding to the 8‐br‐cGMP puff with increased spiking activity (14/164 RGCs) are thus presumed photoreceptor‐driven OFF RGCs (Supporting Information Fig. S6D), whereas those exhibiting a decrease in activity (8/164 RGCs) after the puff are presumed photoreceptor‐driven ON RGCs (Supporting Information Fig. S6E). GABA signaling emerges early during central nervous system (CNS) development, it is then depolarising because of the developmentally regulated low expression of chloride exporters 49. Therefore, GABA can even induce spiking (16/164 RGCs, Supporting Information Fig. S6F) or reduce (12/164 RGCs, Supporting Information Fig. S6G) in RGCs. Hence, responses to GABA puffs are indicative of emerging functional neural networks.

Collectively, our transcriptional and immunohistochemical analysis suggest that the method used for organoid formation and maintenance plays an important role in the generation of retinal organoids and their maturity. Importantly, this integrated analysis indicates the mechanical method of EB formation, supplemented with Y27632 for the first 48 hours of differentiation and linked to stationary maintenance conditions to be the most optimal for generating consistently laminated retinal organoids that are electrophysiologically responsive and closer to developing human fetal retina in gene expression profile.

## Discussion

This study examines whether the methods used to generate EBs at the onset of differentiation have an impact on the retinal differentiation potential of the resulting organoid cultures. During week 5 of differentiation, while the processes associated with the early stages of retinal histogenesis are occurring, transcriptional analysis revealed that retinal organoids clustered more strongly by cell type than by derivation method. Cell line‐specific differences in the expression of several key retinal lineage markers was observed, whereas no significant differences were observed in gene expression across the varying methods of organoid generation. To determine the similitude of in vitro‐generated retinal organoids to native retinal tissue, we examined differentially expressed genes across retinal organoids generated by each method compared with embryonic and fetal human eyes, which revealed that 5‐week‐old organoids aligned more closely with human embryonic retina (4–6.8 PCW). Of all generation methods used, organoid cultures generated under mechanical and static conditions showed the greatest similarity to both embryonic and fetal retinal samples. This data consolidates previous work from our group, looking at the transcriptional profile of retinal organoids generated by the Sasai method 11 at week 5 of differentiation 26.

Comparison of 5‐week‐old organoids with fetal retina (8–18 PCW) showed greater differential gene expression, as expected given the time gap between these developmental stages. Nonetheless, organoid cultures generated by mechanical means, both with and without ROCK inhibition, exhibited the smallest difference in expression compared with fetal retina, indicating this method to be the most efficient in generating retinal organoids with a transcriptional profile resembling native fetal retinal tissue.

Although transcriptional analysis did not identify significant differences during early retinal differentiation between methods, immunohistochemical analysis of organoids did indicate differences in the composition of organoids at week 5. Across all cell lines, laminated retinal neuroepithelium was most consistently observed in cultures derived using the mechanical approach with ROCK inhibition under static conditions. Retinal tissue was composed of retinal progenitor cells, apically positioned photoreceptors, interneurons medially, and retinal ganglion cells at the basal aspect. Developing Müller glia and immunoreactivity for synaptic markers were also observed, indicating that synaptic connections were already being established in the developing outer plexiform and ONLs.

Longer‐term retinal differentiation revealed new insights into the longevity of organoids in culture. Retinal neuroepithelium is visually distinctive and can be easily observed by phase microscopy, as well as confirmed by fixing, sectioning, and performing immunofluorescent histochemistry. Using this approach, we monitored the viability of retinal organoids cultured under long‐term differentiation for up to 22 weeks. Human iPSC cultures generated by enzymatic means (under both static and shaking conditions) were unable to develop to this time point, whereas enzymatically generated hESC‐organoids under static conditions were more stable and remained viable for inclusion in late stage analysis. Interestingly, while at early stages of differentiation retinal organoids clustered more strongly by cell line than by method, at later stages of differentiation (19–21 weeks in vitro) organoids clustered more strongly by their culture maintenance conditions. Pearson correlation analysis of 96 marker genes indicated that organoids maintained under static conditions were more similar to fetal retina than organoids maintained under shaking conditions, and static organoids demonstrated a more advanced retinal maturation expression profile. Retinal organoids grown under shaking conditions exhibited higher levels of photoreceptor precursor markers, whereas static cultures exhibited higher levels of mature retinal marker expression, indicating that shaking cultures may enhance cellular proliferation but not overall maturation, with static cultures more closely resembling fetal retina at this later differentiation stage.

These results were confirmed following immunohistochemical analysis of organoids on weeks 12–13, 17, and 21 of differentiation. Laminated retinal tissue displaying an ONL containing impressive radially aligned photoreceptors featuring advanced morphological maturity were observed in static cultures (both Mech and 96V), whereas large areas of disorganized, less morphologically mature photoreceptors were observed in shaking cultures. The most advanced retinal neuroepithelium with correctly positioned retinal phenotypes was observed in cultures generated by the M&Y_St approach. Not only did developing photoreceptors generated under this condition display immunoreactivity for mature rod and cone markers and develop advanced photoreceptor morphology; TEM analysis of the ultrastructure of these cells allowed the visualization of inner segments, connecting cilium and rudimentary outer segments with clearly discernible membrane discs.

Our observations of retinal differentiation and development of photoreceptor ultrastructure observed in M&Y_St cultures led us to ask whether these organoids might respond to light. Our electrophysiological analysis revealed that high intensity pulsed white light stimulation of 21‐week‐old organoids induced increased spiking activity in photoreceptor‐driven ON retinal ganglion cells, and decreased spiking activity in photoreceptor‐driven OFF retinal ganglion cells. These data indicate that retinal organoids can develop functional neural networks in vitro that act as a functional syncytium and are capable of responding to light stimuli.

In mechanically generated cultures, we observed that treatment with ROCK inhibitor enhanced the overall yield of retinal tissue. Static cultures, in particular, gave rise to very few organoids in the absence of ROCK inhibitor. Not only does ROCK inhibition promote the survival of human pluripotent stem cells upon dissociation 30, 31, the effects of ROCK inhibition on the actin cytoskeleton and the role of the cytoskeleton in the process of tissue self‐organization has also been documented 30, 32, 35, 50, 51, 52, 53, and could be a reason for the enhanced retinal formation in our experiments. Lamas et al. found that ROCK*i* enhanced the proliferation of motor neuron progenitors derived from hESCs and hiPSCs, meaning that the effect of ROCK*i* in neural cultures acts not only to prevent cell death following cellular dissociation, but may also act to promote the expansion of the neural population 54. The enhancement of neurosphere yield, neural crest progenitor, and neural progenitor formation from hESCs under ROCK*i* has also been reported by others 55, 56, 57. ROCK*i*‐induced proliferation of ciliary epithelial retinal progenitors in situ has also been demonstrated in the mouse 58. Furthermore, ROCK*i* can prevent the retraction of adult salamander rod axon terminals/pedicles and increased the number of cone processes and varicosities upon primary culture 59. The effects of ROCK*i* on global gene expression and genomic stability clearly deserve further study.

## Conclusion

Our data show that the method of EB formation and mode of organoid culture maintenance have an impact on in vitro retinal organogenesis. Collectively, our analysis indicates that a mechanical approach to EB generation combined with early ROCK inhibition, followed by organoid maintenance under stationary culture conditions through differentiation is the most efficient approach to consistently produce laminated retinal organoids. The resulting retinal tissue contains each of the key retinal phenotypes, is responsive to light, cGMP and GABA, and most closely resembles human fetal retina compared with organoids derived using alternative derivation methods. This has implications for the mass production of physiologically relevant tissue for use in ophthalmic research and development.

## Author Contributions

C.B.M.: conception and design, collection and assembly of data, data analysis and interpretation, manuscript writing, final approval of manuscript; J.C.: design, collection and assembly of data, data analysis and interpretation, final approval of manuscript; R.Q.: assembly of data, data analysis and interpretation, final approval of manuscript; G.H.: design, collection and assembly of data, data analysis and interpretation, manuscript writing, final approval of manuscript; B.D., D.Z., M.F., K.W.: collection and assembly of data, final approval of manuscript; E.S.: design, collection and assembly of data, data analysis and interpretation, manuscript writing, final approval of manuscript; M.L.: conception and design, collection and assembly of data, data analysis and interpretation, manuscript writing, final approval of manuscript, financial support.

## Disclosure of Potential Conflicts of Interest

The authors indicated no potential conflicts of interest.

## Supporting information


**Supplementary Figure 1 The effect of ROCK inhibition on early embryoid body morphology. (A‐C)** The addition of Y‐27632 (&Y) during the first 48 hours of differentiation in enzymatically‐generated cultures **(B)** resulted in increased EB size compared to EBs generated without Y (No Y) **(A)**. **(C)** Doubling the concentration of Y‐27632 increased EB size further. Scale bars = 200 μm.

**Supplementary Figure 2 The morphology of retinal organoids generated by each method across three cell lines on day 35 of differentiation. (A‐I)** Representative examples of organoids observed on day 35 of differentiation, across all lines and all methods tested. Retinal organoids exhibit a typical morphology which is clearly identifiable in in vitro culture, which was most consistently achieved using a mechanical stationary approach **(A‐C)**. Retinal organoids were able to form at different frequencies under almost all conditions tested, but adopting certain methods of EB formation at the onset of differentiation caused the morphology of the overall culture to be greatly altered. Of note, shaking mechanical cultures gave rise to EBs with significantly flattened edges **(A)**, and 96 well plate generated cultures gave rise to EBs which were folded and sometimes highly convoluted **(G, H)** compared with other methods. All scale bars = 200 μm.

**Supplementary Figure 3 Micrographs demonstrating the internal cytoarchitecture of embryoid bodies depending on the method used at the onset of differentiation. (A, B)** M&Y Stationary cultures generated retinal organoids with clearly developing outer (“O” or “ONL”) and inner (“I” or “INL”) neuroblastic layers. **(C, D)** M&Y shaking cultures generated EBs with a disorganized interior and intermittent regions of defined, flattened neuroepithelium at the exterior. (E, F, G) EB generation using 96 well plates routinely gave rise to EBs consisting of a body containing neural rosettes (asterisks) from which grew fine extensions akin to those observed in pulmonary organoids (arrows). Scale bars = A, C, E‐G = 200 μm, B = 50 μm, D = 100 μm.

**Supplementary Figure 4 Differences observed in EB morphology resulting from the shape of the 96 well plate used from the outset.** Neuroepithelium was observed in EBs generated from hESCs using both U‐ and V‐shaped 96 well plates if kept under stationary conditions **(A, B)**, but not under shaking conditions **(E, F)**. **(C, G)** U‐shaped wells readily gave rise to AD3‐derived EBs with clearly identifiable retinal neuroepithelium under both stationary and shaking conditions, whilst those generated using V‐shaped wells **(D, H)** displayed thinner, more convoluted neuroepithelium. Scale bars; A = 200um, all images display the same magnification.

**Supplementary Figure 5 Cellular morphology and ultrastructure of “M&Y_St” organoid‐derived photoreceptors at later stages of differentiation. (A‐C, D*i, ii*)** AD3‐derived photoreceptors on day 150 of differentiation were immunopositive for pan‐photoreceptor marker Recoverin **(A)**, cone‐specific Opsin Blue **(B)** and rod‐specific gαt1 **(C)**. **(B)** Some punctate Bassoon reactivity was observed in the outer nuclear layer, indicative of ribbon synapse formation. **(D*i, ii*)** AD3‐derived and **(D*iii*)** H9‐derived retinal ganglion cells immunopositive for Smi32, **(D*iii*)** RBPMS and HuCD on day 150. **(E‐L)** TEM micrographs showing inner segments, connecting cilium and outer segments in photoreceptors developing within AD3‐derived retinal organoids on day 170 of differentiation and **(I‐L)** outer segments shown at higher magnification displaying the appearance of developing membrane discs. Scale bars; A, B = 20 μm, C, D = 50 μm, E = 2 μm, F, H, J‐L = 500 nm, G, I = 1 μm.

**Supplementary Figure 6 Spiking activity recorded from presumed RGCs of 3D retinas derived from the hESC (H9) line at day 150 of differentiation using the M&Y_St method. A, B)** Spike raster plots (SRPs) from RGCs that showed a 25% increase **(A)** and decrease **(B)** in spiking activity during pulsed white light (WLP, see methods). In SRPs, each vertical bar indicates the time stamp of a spike, where each row represents a different RGC. The left half illustrates the activity before stimulus onset and, separated by the red line, the right half the activity when exposed to WLP. **C)** SRPs from intrinsically photosensitive RGCs that showed a 25% increase in spiking activity during constant blue light (BLC, see methods). **D, E)** SRPs from RGCs that showed a 25% increase **(D)** and decrease **(E)** in spiking activity after puffing 8‐br‐cGMP (final concentration 100 μM). **F, G)** SRPs from RGCs that showed a 25% increase **(F)** and decrease **(G)** in spiking activity after puffing GABA (final concentration 125 μM).

**Supplementary Table 1** Antibodies used for immunohistochemistry.

**Supplementary Table 2** Summary of TaqMan qPCR arrays.

**Supplementary Table 3** Summary of differentially expressed genes between retinal organoids generated from all three pluripotent stem cell lines at week 5 of differentiation.

**Supplementary Table 4** Summary of differentially expressed genes between retinal organoids generated with different methods at week 5 of differentiation and human embryonic (hEye) and fetal retina (hRet) samples.

**Supplementary Table 5** Summary of differentially expressed genes between retinal organoids generated under shaking and static conditions at 19–21 week of differentiation and foetal (hRet) samples.

## References

[1] Hirami Y , Osakada F , Takahashi K et al. Generation of retinal cells from mouse and human induced pluripotent stem cells. Neurosci Lett 2009;458:126–131. 19379795 10.1016/j.neulet.2009.04.035

[2] Lamba DA , Karl MO , Ware CB et al. Efficient generation of retinal progenitor cells from human embryonic stem cells. Proc Natl Acad Sci USA 2006;103:12769–12774. 16908856 10.1073/pnas.0601990103 PMC1568922

[3] Mellough CB , Sernagor E , Moreno‐Gimeno I et al. Efficient stage‐specific differentiation of human pluripotent stem cells toward retinal photoreceptor cells. Stem Cells 2012;30:673–686. 22267304 10.1002/stem.1037

[4] Osakada F , Ikeda H , Mandai M et al. Toward the generation of rod and cone photoreceptors from mouse, monkey and human embryonic stem cells. Nat Biotechnol 2008;26:215–224. 18246062 10.1038/nbt1384

[5] Osakada F , Jin ZB , Hirami Y et al. In vitro differentiation of retinal cells from human pluripotent stem cells by small‐molecule induction. J Cell Sci 2009;122:3169–3179. 19671662 10.1242/jcs.050393

[6] Meyer JS , Shearer RL , Capowski EE et al. Modeling early retinal development with human embryonic and induced pluripotent stem cells. Proc Natl Acad Sci USA 2009;106:16698–16703. 19706890 10.1073/pnas.0905245106 PMC2757802

[7] Collin J , Mellough CB , Dorgau B et al. Using zinc finger nuclease technology to generate CRX‐reporter human embryonic stem cells as a tool to identify and study the emergence of photoreceptors precursors during pluripotent stem cell differentiation. Stem Cells 2016;34:311–321. 26608863 10.1002/stem.2240 PMC4832345

[8] Mellough CB , Collin J , Khazim M et al. IGF‐1 signaling plays an important role in the formation of three‐dimensional laminated neural retina and other ocular structures from human embryonic stem cells. Stem Cells 2015;33:2416–2430. 25827910 10.1002/stem.2023 PMC4691326

[9] Phillips MJ , Wallace KA , Dickerson SJ et al. Blood‐derived human iPS cells generate optic vesicle‐like structures with the capacity to form retinal laminae and develop synapses. Invest Ophthalmol Vis Sci 2012;53:2007–2019. 22410558 10.1167/iovs.11-9313 PMC3648343

[10] Eiraku M , Takata N , Ishibashi H et al. Self‐organizing optic‐cup morphogenesis in three‐dimensional culture. Nature 2011;472:51–56. 21475194 10.1038/nature09941

[11] Nakano T , Ando S , Takata N et al. Self‐formation of optic cups and storable stratified neural retina from human ESCs. Cell Stem Cell 2012;10:771–785. 22704518 10.1016/j.stem.2012.05.009

[12] Hiler D , Chen X , Hazen J et al. Quantification of retinogenesis in 3D cultures reveals epigenetic memory and higher efficiency in iPSCs derived from rod photoreceptors. Cell Stem Cell 2015;17:101–115. 26140606 10.1016/j.stem.2015.05.015 PMC4547539

[13] Kaewkhaw R , Kaya KD , Brooks M et al. Transcriptome dynamics of developing photoreceptors in three‐dimensional retina cultures recapitulates temporal sequence of human cone and rod differentiation revealing cell surface markers and gene networks. Stem Cells 2015;33:3504–3518. 26235913 10.1002/stem.2122 PMC4713319

[14] Kuwahara A , Ozone C , Nakano T et al. Generation of a ciliary margin‐like stem cell niche from self‐organizing human retinal tissue. Nat Commun 2015;6:6286. 25695148 10.1038/ncomms7286

[15] Reichman S , Terray A , Slembrouck A et al. From confluent human iPS cells to self‐forming neural retina and retinal pigmented epithelium. Proc Natl Acad Sci USA 2014;111:8518–8523. 24912154 10.1073/pnas.1324212111 PMC4060726

[16] Volkner M , Zschätzsch M , Rostovskaya M et al. Retinal organoids from pluripotent stem cells efficiently recapitulate retinogenesis. Stem Cell Rep 2016;6:525–538. 10.1016/j.stemcr.2016.03.001 PMC4834051 27050948

[17] Wang XB , Xiong K , Lin C et al. New medium used in the differentiation of human pluripotent stem cells to retinal cells is comparable to fetal human eye tissue. Biomaterials 2015;53:40–49. 25890705 10.1016/j.biomaterials.2015.02.065

[18] Zhong X , Gutierrez C , Xue T et al. Generation of three‐dimensional retinal tissue with functional photoreceptors from human iPSCs. Nat Commun 2014;5:4047. 24915161 10.1038/ncomms5047 PMC4370190

[19] Zhou S , Flamier A , Abdouh M et al. Differentiation of human embryonic stem cells into cone photoreceptors through simultaneous inhibition of BMP, TGFβ and Wnt signaling. Development 2015;142:3294–3306. 26443633 10.1242/dev.125385

[20] Hayashi R , Ishikawa Y , Sasamoto Y et al. Co‐ordinated ocular development from human iPS cells and recovery of corneal function. Nature 2016;531:376–380. 26958835 10.1038/nature17000

[21] Jin ZB , Okamoto S , Osakada F et al. Modeling retinal degeneration using patient‐specific induced pluripotent stem cells. PLoS One 2011;6:e17084. 21347327 10.1371/journal.pone.0017084 PMC3037398

[22] Meyer JS , Howden SE , Wallace KA et al. Optic vesicle‐like structures derived from human pluripotent stem cells facilitate a customized approach to retinal disease treatment. Stem Cells 2011;29:1206–1218. 21678528 10.1002/stem.674 PMC3412675

[23] Deng WL , Gao ML , Lei XL et al. Gene correction reverses ciliopathy and photoreceptor loss in IPSC‐derived retinal organoids from retinitis pigmentosa patients. Stem Cell Rep 2018;10:1267–1281. 10.1016/j.stemcr.2018.02.003 PMC5998840 29526738

[24] Lowe A , Harris R , Bhansali P et al. Intercellular adhesion‐dependent cell survival and rock‐regulated actomyosin‐driven forces mediate self‐formation of a retinal organoid. Stem Cell Rep 2016;6:743–756. 10.1016/j.stemcr.2016.03.011 PMC4939656 27132890

[25] Parfitt DA , Lane A , Ramsden CM et al. Identification and correction of mechanisms underlying inherited blindness in human iPSC‐derived optic cups. Cell Stem Cell 2016;18:769–781. 27151457 10.1016/j.stem.2016.03.021 PMC4899423

[26] Hallam D , Hilqen G , Dorgau B et al. Human‐induced pluripotent stem cells generate light responsive retinal organoids with variable and nutrient‐dependent efficiency. Stem Cells 2018;36:1535–1551. 30004612 10.1002/stem.2883 PMC6392112

[27] Phillips MJ , Perez ET , Martin JM et al. Modeling human retinal development with patient‐specific induced pluripotent stem cells reveals multiple roles for visual system homeobox 2. Stem Cells 2014;32:1480–1492. 24532057 10.1002/stem.1667 PMC4037340

[28] Tucker BA , Mullins RF , Streb LM et al. Patient‐specific iPSC‐derived photoreceptor precursor cells as a means to investigate retinitis pigmentosa. eLife 2013;2:e00824. 10.7554/eLife.00824 PMC3755341 23991284

[29] Buskin A , Zhu L , Chichagova V et al. Splicing factor PRPF31 retinitis pigmentosa (RP11) is caused by disrupted alternative splicing programmes for genes implicated in pre‐mRNA splicing, cellular adhesion and ciliogenesis. Nature Commun 2018;9:4234. 10.1038/s41467-018-06448-y PMC6185938 30315276

[30] Ohgushi M , Matsumura M , Eiraku M et al. Molecular pathway and cell state responsible for dissociation‐induced apoptosis in human pluripotent stem cells. Cell Stem Cell 2010;7:225–239. 20682448 10.1016/j.stem.2010.06.018

[31] Watanabe K , Ueno M , Kamiya D et al. A ROCK inhibitor permits survival of dissociated human embryonic stem cells. Nat Biotechnol 2007;25:681–686. 17529971 10.1038/nbt1310

[32] Chen G , Hou Z , Gulbranson DR et al. Actin‐myosin contractility is responsible for the reduced viability of dissociated human embryonic stem cells. Cell Stem Cell 2010;7:240–248. 20682449 10.1016/j.stem.2010.06.017 PMC2916864

[33] Yanai A , Laver C , Joe AW et al. Efficient production of photoreceptor precursor cells from human embryonic stem cells. Methods Mol Biol 2016;1307:357–369. 24301073 10.1007/7651_2013_57

[34] Shirai H , Mandai M , Matsushita K et al. Transplantation of human embryonic stem cell‐derived retinal tissue in two primate models of retinal degeneration. Proc Natl Acad Sci USA 2016;113:E81–E90. 26699487 10.1073/pnas.1512590113 PMC4711854

[35] Compagnucci C , Baressi S , Petrini S et al. Rho kinase inhibition is essential during in vitro neurogenesis and promotes phenotypic rescue of human induced pluripotent stem cell‐derived neurons with oligophrenin‐1 loss of function. Stem Cells Translational Medicine 2016;5:860–869. 27160703 10.5966/sctm.2015-0303 PMC4922854

[36] Kamishibahara Y , Kawaguchi H , Shimizu N . Rho kinase inhibitor Y‐27632 promotes neuronal differentiation in mouse embryonic stem cells via phosphatidylinositol 3‐kinase. Neurosci Lett 2016;615:44–49. 26797580 10.1016/j.neulet.2016.01.022

[37] Nakamura M , Kamishibahara Y , Kitazawa A et al. Differentiation patterns of mouse embryonic stem cells and induced pluripotent stem cells into neurons. Cytotechnology 2016;68:409–417. 25354731 10.1007/s10616-014-9792-2 PMC4846643

[38] Chunbo Yang YX , Min Y , Lee D et al. Induced pluripotent stem cell modelling of HLHS underlines the contribution of dysfunctional NOTCH signalling to impaired cardiogenesis. Hum Mol Genet 2017;26:3031–3045. 28521042 10.1093/hmg/ddx140 PMC5886295

[39] Jiang Y , Habibollah S , Tilgner K et al. An induced pluripotent stem cell model of hypoplastic left heart syndrome (HLHS) reveals multiple expression and functional differences in HLHS‐derived cardiac myocytes. Stem Cells Translational Medicine 2014;3:416–423. 24591732 10.5966/sctm.2013-0105 PMC3973710

[40] Melguizo‐Sanchis D , Xu Y , Taheem D et al. iPSC modeling of severe aplastic anemia reveals impaired differentiation and telomere shortening in blood progenitors. Cell Death Dis 2018;9:128. 29374141 10.1038/s41419-017-0141-1 PMC5833558

[41] Eiraku M , Watanabe K , Matsuo‐Takasaki M et al. Self‐organized formation of polarized cortical tissues from escs and its active manipulation by extrinsic signals. Cell Stem Cell 2008;3:519–532. 18983967 10.1016/j.stem.2008.09.002

[42] Hilgen G , Pirmoradian S , Pamplona D et al. Pan‐retinal characterisation of light responses from ganglion cells in the developing mouse retina. Sci Rep 2017;7:42330. 28186129 10.1038/srep42330 PMC5301206

[43] Muthmann JO , Amin H , Sernagor E et al. Spike detection for large neural populations using high density multielectrode arrays. Front Neuroinform 2015;9:28. 26733859 10.3389/fninf.2015.00028 PMC4683190

[44] Hilgen G , Sorbaro M , Pirmoradian S et al. Unsupervised spike sorting for large‐scale, high‐density multielectrode arrays. Cell Rep 2017;18:2521–2532. 28273464 10.1016/j.celrep.2017.02.038

[45] Mellough CB, Bauer R, Collin J, An integrated transcriptional analysis of the developing human retina. Development 2019;146, pii: dev169474 10.1242/dev.169474 PMC6361134 30696714

[46] Lin B , Wang SW , Masland RH . Retinal ganglion cell type, size, and spacing can be specified independent of homotypic dendritic contacts. Neuron 2004;43:475–485. 15312647 10.1016/j.neuron.2004.08.002

[47] Coombs J , van der List D , Wang GY et al. Morphological properties of mouse retinal ganglion cells. Neuroscience 2006;140:123–136. 16626866 10.1016/j.neuroscience.2006.02.079

[48] Rodriguez AR , de Sevilla Müller LP , Brecha NC . The RNA binding protein RBPMS is a selective marker of ganglion cells in the mammalian retina. J Comp Neurol 2014;522:1411–1443. 24318667 10.1002/cne.23521 PMC3959221

[49] Ben‐Ari Y . Excitatory actions of gaba during development: The nature of the nurture. Nat Rev Neurosci 2002;3:728–739. 12209121 10.1038/nrn920

[50] Ohgushi M , Minaguchi M , Sasai Y . Rho‐signaling‐directed YAP/TAZ activity underlies the long‐term survival and expansion of human embryonic stem cells. Cell Stem Cell 2015;17:448–461. 26321201 10.1016/j.stem.2015.07.009

[51] Gomes ER , Jani S , Gundersen GG . Nuclear movement regulated by Cdc42, MRCK, myosin, and actin flow establishes MTOC polarization in migrating cells. Cell 2005;121:451–463. 15882626 10.1016/j.cell.2005.02.022

[52] Heynen SR , Meneau I , Caprara C et al. CDC42 is required for tissue lamination and cell survival in the mouse retina. PLoS One 2013;8:e53806. 23372671 10.1371/journal.pone.0053806 PMC3553133

[53] Compagnucci C , Piermarini E , Sferra A et al. Cytoskeletal dynamics during in vitro neurogenesis of induced pluripotent stem cells (iPSCs). Mol Cell Neurosci 2016;77:113–124. 27756615 10.1016/j.mcn.2016.10.002

[54] Lamas NJ , Johnson‐Kerner B , Roybon L et al. Neurotrophic requirements of human motor neurons defined using amplified and purified stem cell‐derived cultures. PLoS One 2014;9:e110324. 25337699 10.1371/journal.pone.0110324 PMC4206291

[55] Chaddah R , Arntfield M , Runciman S et al. Clonal neural stem cells from human embryonic stem cell colonies. J Neurosci 2012;32:7771–7781. 22674254 10.1523/JNEUROSCI.3286-11.2012 PMC6620945

[56] Kim K , Ossipova O , Sokol SY . Neural crest specification by inhibition of the ROCK/Myosin II pathway. Stem Cells 2015;33:674–685. 25346532 10.1002/stem.1877 PMC4428345

[57] Rungsiwiwut R , Manolertthewan C , Numchaisrika P et al. The ROCK inhibitor Y‐26732 enhances the survival and proliferation of human embryonic stem cell‐derived neural progenitor cells upon dissociation. Cells Tissues Organs 2013;198:127–138. 24158103 10.1159/000354031

[58] Del Debbio CB , Santos MF , Yan CY et al. Rho GTPases control ciliary epithelium cells proliferation and progenitor profile induction in vivo. Invest Ophthalmol Vis Sci 2014;55:2631–2641. 24692128 10.1167/iovs.13-13162

[59] Fontainhas AM , Townes‐Anderson E . RhoA and its role in synaptic structural plasticity of isolated salamander photoreceptors. Invest Ophthalmol Vis Sci 2008;49:4177–4187. 18503000 10.1167/iovs.07-1580

