# Supplementary figures and images for: Systematic Comparison of Retinal Organoid Differentiation from Human Pluripotent Stem Cells Reveals Stage Specific, Cell Line, and Methodological Differences
Source: Stem Cells Transl Med. 2019 Mar 27;8(7):694–706. doi: 10.1002/sctm.18-0267 (PMC6591558; doi:10.1002/sctm.18-0267)

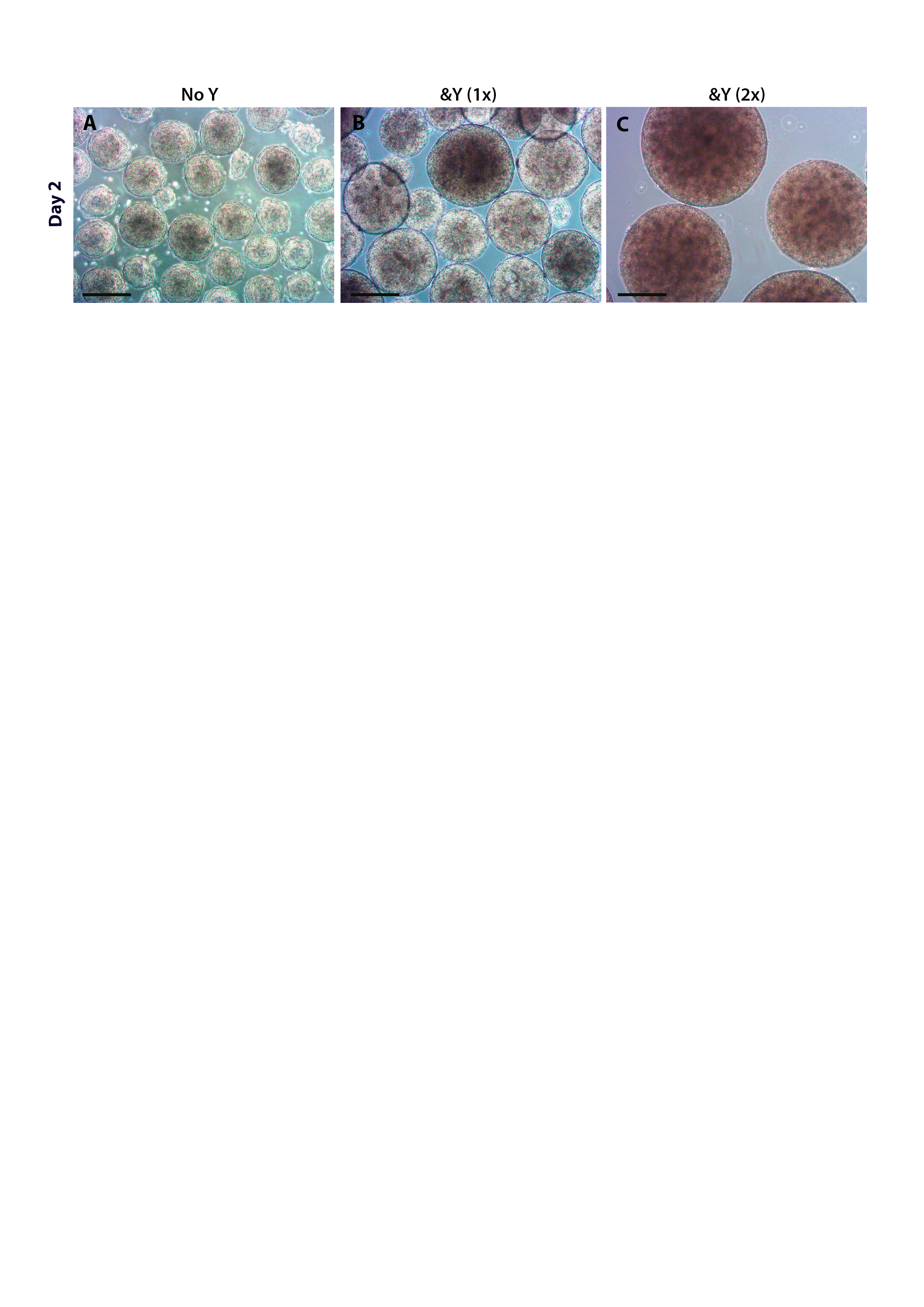

Supplement: Supplementary file 1 — Supplementary Figure 1 The effect of ROCK inhibition on early embryoid body morphology. (A‐C) The addition of Y‐27632 (&Y) during the first 48 hours of differentiation in enzymatically‐generated cultures (B) resulted in increased EB size compared to EBs generated without Y (No Y) (A). (C) Doubling the concentration of Y‐27632 increased EB size further. Scale bars = 200 μm. [file SCT3-8-694-s001.tif]

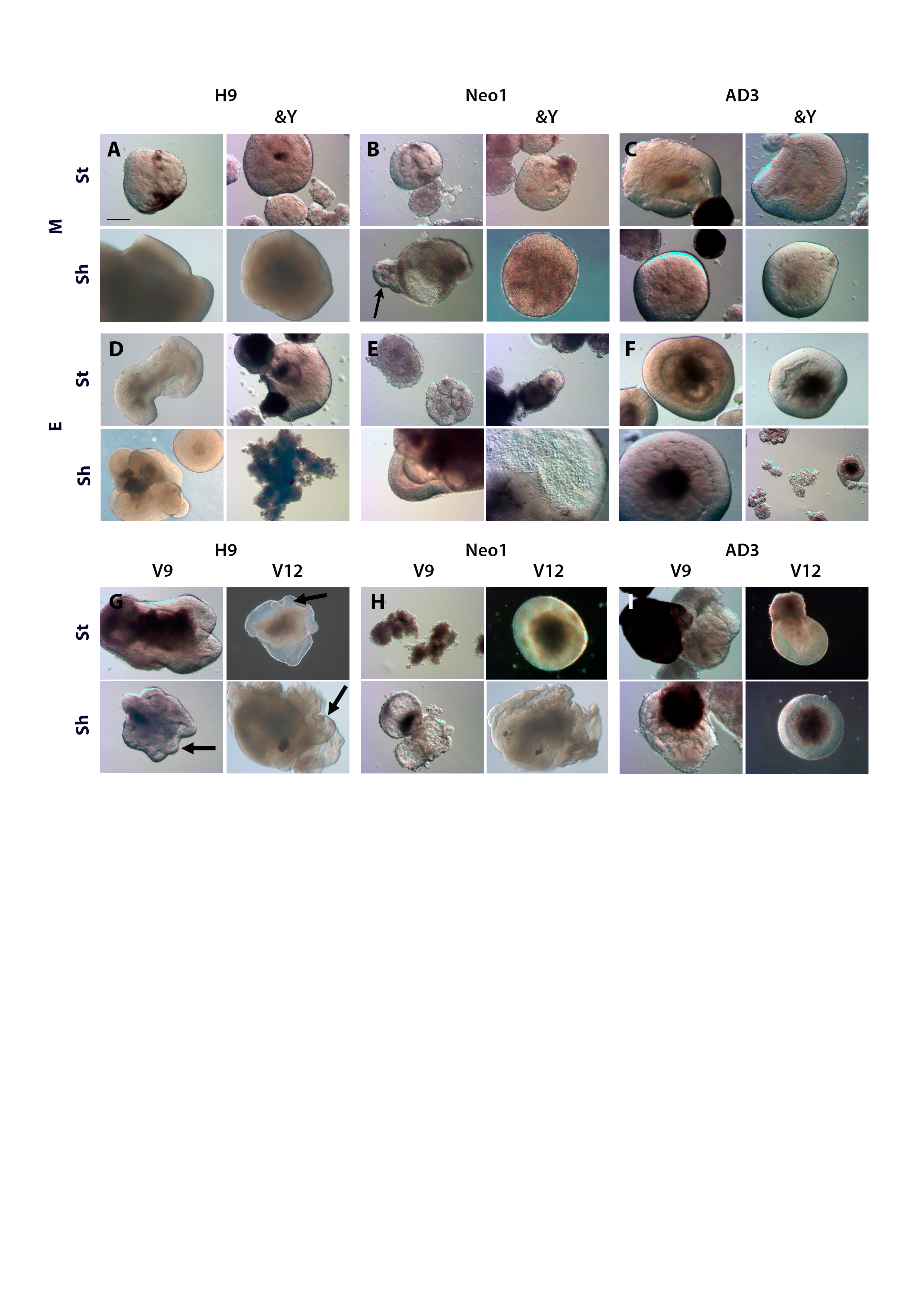

Supplement: Supplementary file 2 — Supplementary Figure 2 The morphology of retinal organoids generated by each method across three cell lines on day 35 of differentiation. (A‐I) Representative examples of organoids observed on day 35 of differentiation, across all lines and all methods tested. Retinal organoids exhibit a typical morphology which is clearly identifiable in in vitro culture, which was most consistently achieved using a mechanical stationary approach (A‐C). Retinal organoids were able to form at different frequencies under almost all conditions tested, but adopting certain methods of EB formation at the onset of differentiation caused the morphology of the overall culture to be greatly altered. Of note, shaking mechanical cultures gave rise to EBs with significantly flattened edges (A), and 96 well plate generated cultures gave rise to EBs which were folded and sometimes highly convoluted (G, H) compared with other methods. All scale bars = 200 μm. [file SCT3-8-694-s002.tif]

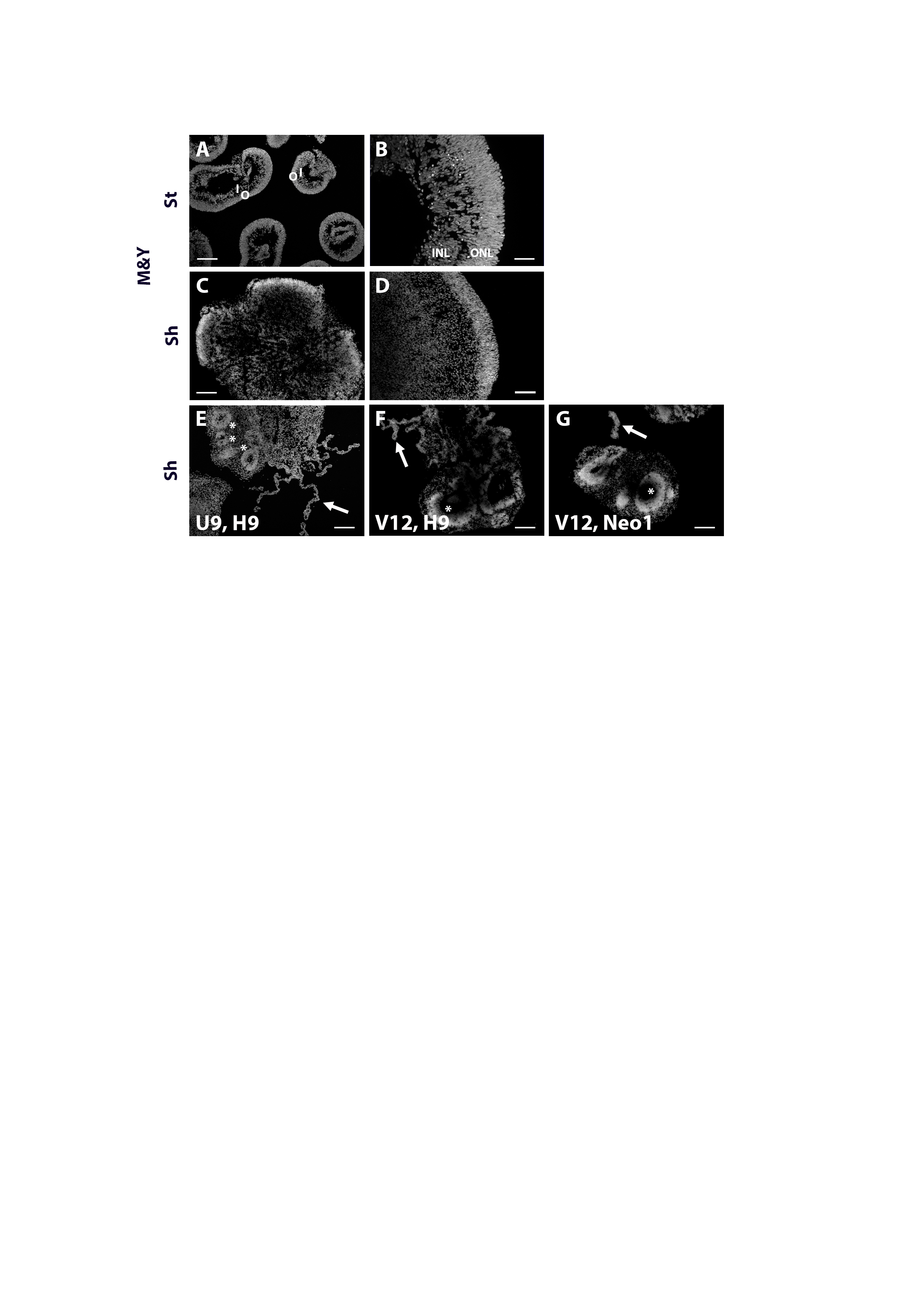

Supplement: Supplementary file 3 — Supplementary Figure 3 Micrographs demonstrating the internal cytoarchitecture of embryoid bodies depending on the method used at the onset of differentiation. (A, B) M&Y Stationary cultures generated retinal organoids with clearly developing outer (“O” or “ONL”) and inner (“I” or “INL”) neuroblastic layers. (C, D) M&Y shaking cultures generated EBs with a disorganized interior and intermittent regions of defined, flattened neuroepithelium at the exterior. (E, F, G) EB generation using 96 well plates routinely gave rise to EBs consisting of a body containing neural rosettes (asterisks) from which grew fine extensions akin to those observed in pulmonary organoids (arrows). Scale bars = A, C, E‐G = 200 μm, B = 50 μm, D = 100 μm. [file SCT3-8-694-s003.tif]

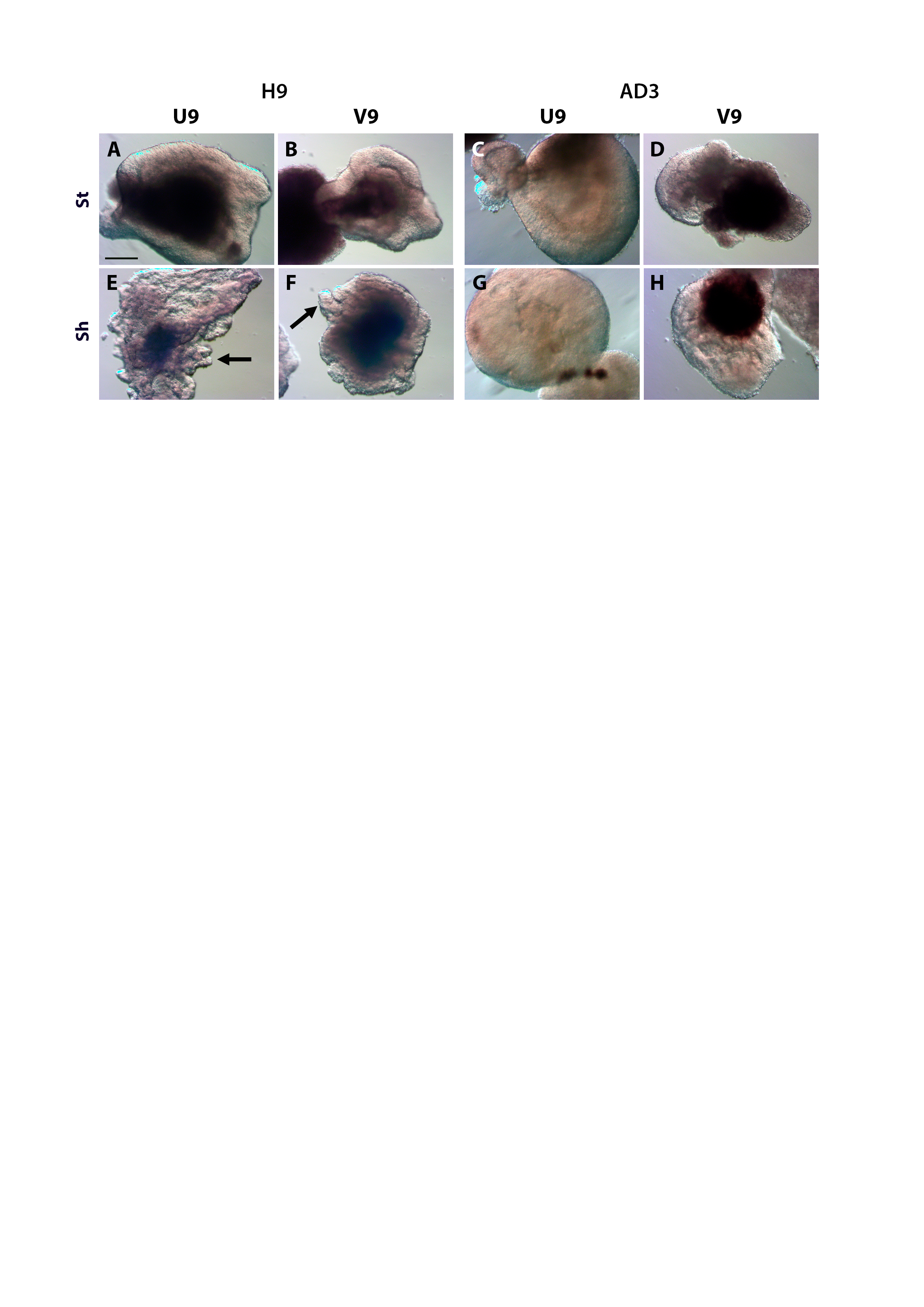

Supplement: Supplementary file 4 — Supplementary Figure 4 Differences observed in EB morphology resulting from the shape of the 96 well plate used from the outset. Neuroepithelium was observed in EBs generated from hESCs using both U‐ and V‐shaped 96 well plates if kept under stationary conditions (A, B), but not under shaking conditions (E, F). (C, G) U‐shaped wells readily gave rise to AD3‐derived EBs with clearly identifiable retinal neuroepithelium under both stationary and shaking conditions, whilst those generated using V‐shaped wells (D, H) displayed thinner, more convoluted neuroepithelium. Scale bars; A = 200um, all images display the same magnification. [file SCT3-8-694-s004.tif]

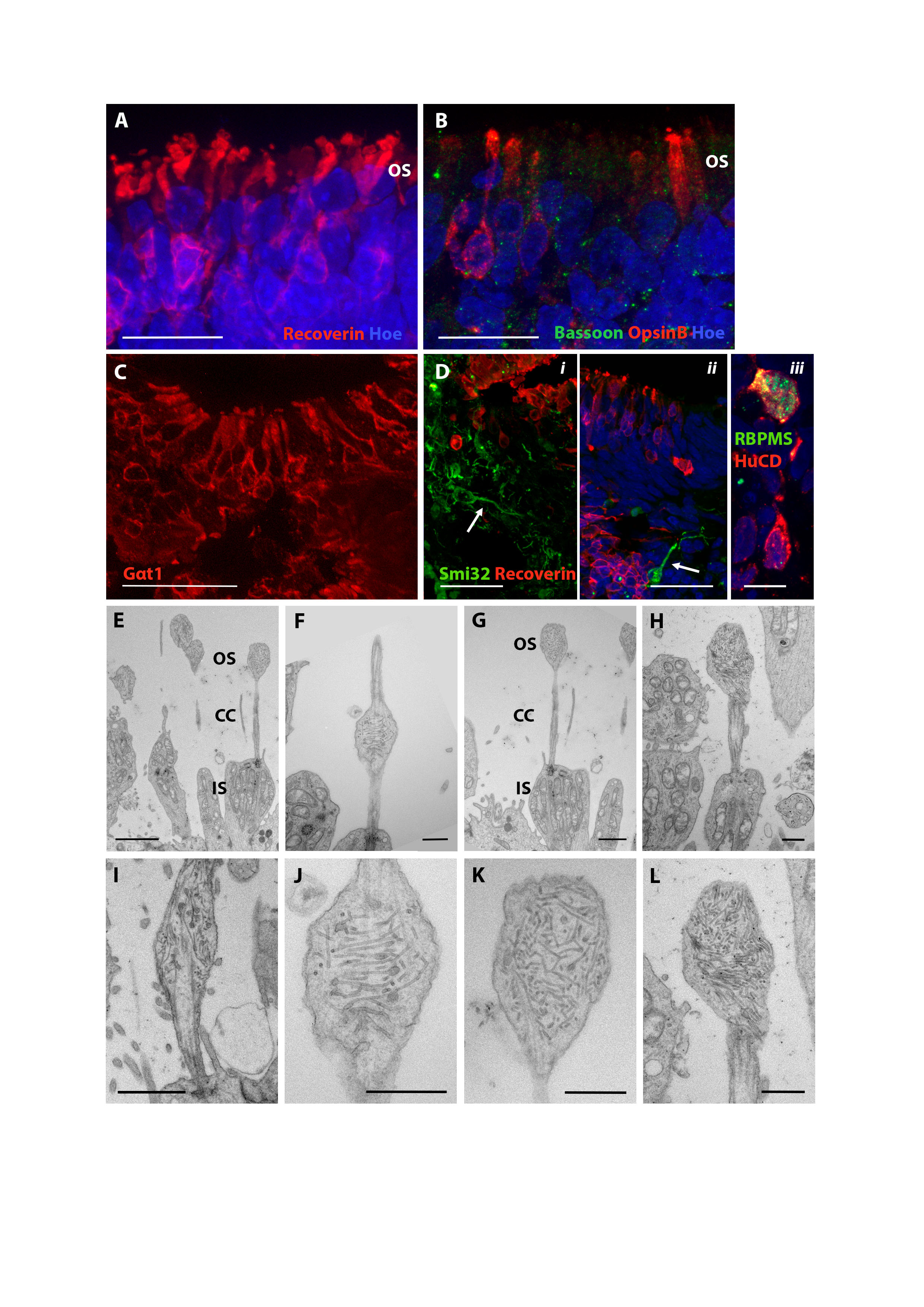

Supplement: Supplementary file 5 — Supplementary Figure 5 Cellular morphology and ultrastructure of “M&Y_St” organoid‐derived photoreceptors at later stages of differentiation. (A‐C, Di, ii) AD3‐derived photoreceptors on day 150 of differentiation were immunopositive for pan‐photoreceptor marker Recoverin (A), cone‐specific Opsin Blue (B) and rod‐specific gαt1 (C). (B) Some punctate Bassoon reactivity was observed in the outer nuclear layer, indicative of ribbon synapse formation. (Di, ii) AD3‐derived and (Diii) H9‐derived retinal ganglion cells immunopositive for Smi32, (Diii) RBPMS and HuCD on day 150. (E‐L) TEM micrographs showing inner segments, connecting cilium and outer segments in photoreceptors developing within AD3‐derived retinal organoids on day 170 of differentiation and (I‐L) outer segments shown at higher magnification displaying the appearance of developing membrane discs. Scale bars; A, B = 20 μm, C, D = 50 μm, E = 2 μm, F, H, J‐L = 500 nm, G, I = 1 μm. [file SCT3-8-694-s005.jpg]

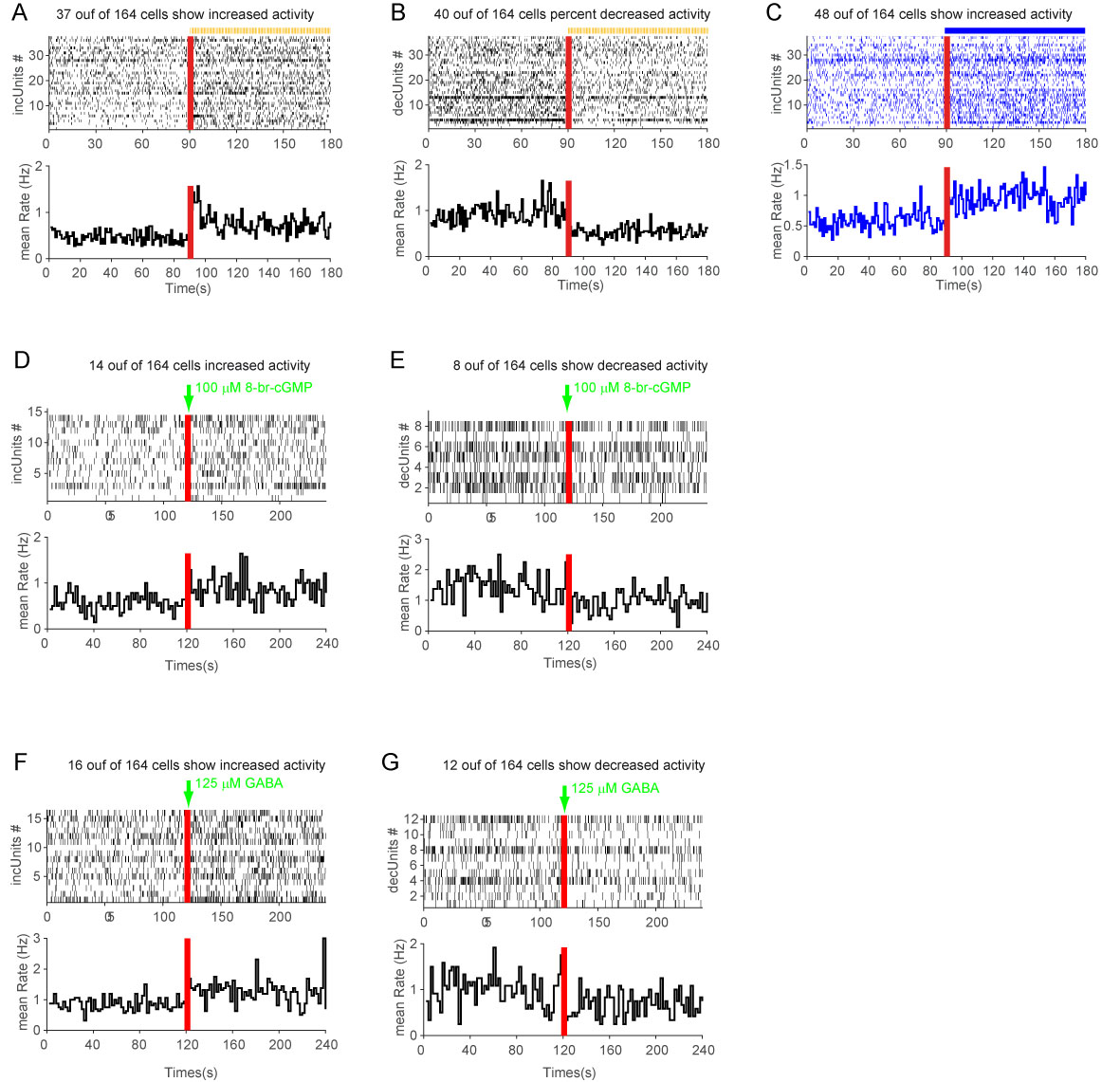

Supplement: Supplementary file 6 — Supplementary Figure 6 Spiking activity recorded from presumed RGCs of 3D retinas derived from the hESC (H9) line at day 150 of differentiation using the M&Y_St method. A, B) Spike raster plots (SRPs) from RGCs that showed a 25% increase (A) and decrease (B) in spiking activity during pulsed white light (WLP, see methods). In SRPs, each vertical bar indicates the time stamp of a spike, where each row represents a different RGC. The left half illustrates the activity before stimulus onset and, separated by the red line, the right half the activity when exposed to WLP. C) SRPs from intrinsically photosensitive RGCs that showed a 25% increase in spiking activity during constant blue light (BLC, see methods). D, E) SRPs from RGCs that showed a 25% increase (D) and decrease (E) in spiking activity after puffing 8‐br‐cGMP (final concentration 100 μM). F, G) SRPs from RGCs that showed a 25% increase (F) and decrease (G) in spiking activity after puffing GABA (final concentration 125 μM). [file SCT3-8-694-s006.tif]
