# Supplementary material for: Systematic Comparison of Retinal Organoid Differentiation from Human Pluripotent Stem Cells Reveals Stage Specific, Cell Line, and Methodological Differences
Source: Stem Cells Transl Med. 2019 Mar 27;8(7):694–706. doi: 10.1002/sctm.18-0267 (PMC6591558; doi:10.1002/sctm.18-0267)
Supplement: Supplementary file 7 — Supplementary Table 1 Antibodies used for immunohistochemistry. [file SCT3-8-694-s007.docx]

| **Antibody Name** | **Catalogue Number** | **Source** | **Dilution** |
| --- | --- | --- | --- |
| Bassoon | VAM-PS003 | StressGen | 1:100 |
| CRALBP | GTX15051 | Genetex | 1:100 |
| Crx (MO2) clone 4G11 | H00001406-M02 | Abnova | 1:200 |
| Gαt1 (K-20) | Sc-389 | Santa Cruz | 1:200 |
| HuCD | A21271 | Invitrogen | 1:200 |
| Opsin blue | AB5407 | Millipore | 1:200 |
| OTX2 | Ab114138 | Abcam | 1:200 |
| RBPMS | 1830-RBPMS | PhosphoSolutions | 1:1000 |
| Recoverin | AB5585 | Millipore | 1:1000 |
| Smi32 | SMI-32R | Covance | 1:100 |
| SOX2 | MAB2018 | R&D | 1:200 |
| Synaptophysin | MAB368 | Millipore | 1:100 |
| Vimentin |  | Gift from Roy Quinlan | 1:400 |
| VSX2 | HPA003436 | Sigma Atlas | 1:200 |
